# Forecasting continued increase in primary and secondary syphilis in the United States, 2024–2033: a state-level multi-model ensemble study

**DOI:** 10.3389/fpubh.2026.1888674

**Published:** 2026-07-24

**Authors:** Qiancheng Ma, Tianzhen Zhang, Dongzhi Lin, Wenyuan Zou, Liuying Lin

**Affiliations:** 1 Department of Community Health, Faculty of Medicine and Health Sciences, Universiti Putra Malaysia, Serdang, Malaysia; 2 School of Nursing and Midwifery, Trinity College Dublin, Dublin, Ireland; 3 School of Information Engineering, Fujian Polytechnic of Water Conservancy and Electric Power, Sanming, China; 4 Department of Nursing, Faculty of Medicine and Health Sciences, Universiti Putra Malaysia, Serdang, Malaysia

**Keywords:** forecasting, machine learning, multi-model ensemble, primary and secondary syphilis, state-level surveillance, United States

## Abstract

**Introduction:**

Primary and secondary (P&S) syphilis burden in the United States has increased substantially in recent years, underscoring the need for forecasting approaches that can inform medium-term public health planning.

**Methods:**

We assembled a state-level panel dataset covering 2003–2023 and evaluated five candidate forecasting models: autoregressive model of order 1 [AR(1)], a state-specific linear trend baseline, Bayesian negative binomial regression, neural network, and Extreme Gradient Boosting (XGBoost). Models were trained using historical state-level surveillance and covariate data and assessed on external holdout data. For the non-Bayesian models, prediction intervals were calibrated using a residual-based calibration approach, whereas Bayesian uncertainty was obtained from the posterior predictive distribution. A final top-3 global ensemble was constructed from the best-performing models.

**Results:**

The final ensemble achieved a holdout weighted absolute percentage error (WAPE) of 20.8%. Under the continuation of the main historical patterns represented in the data, the national burden was projected to increase to approximately 131,736 cases in 2033. Projected cumulative burden was highest in California, Florida, New York, Texas, and Georgia, whereas the fastest relative growth was projected in Iowa, North Dakota, Wisconsin, South Dakota, and Nevada. Diagnostic analyses found no evidence of residual spatial autocorrelation in the final ensemble.

**Discussion:**

This forecasting framework provides a practical approach for projecting state-level syphilis burden in the United States. It may support public health planning by distinguishing states with persistently high projected burden from those with comparatively rapid projected growth.

## 1 Introduction

**Figure 1:**
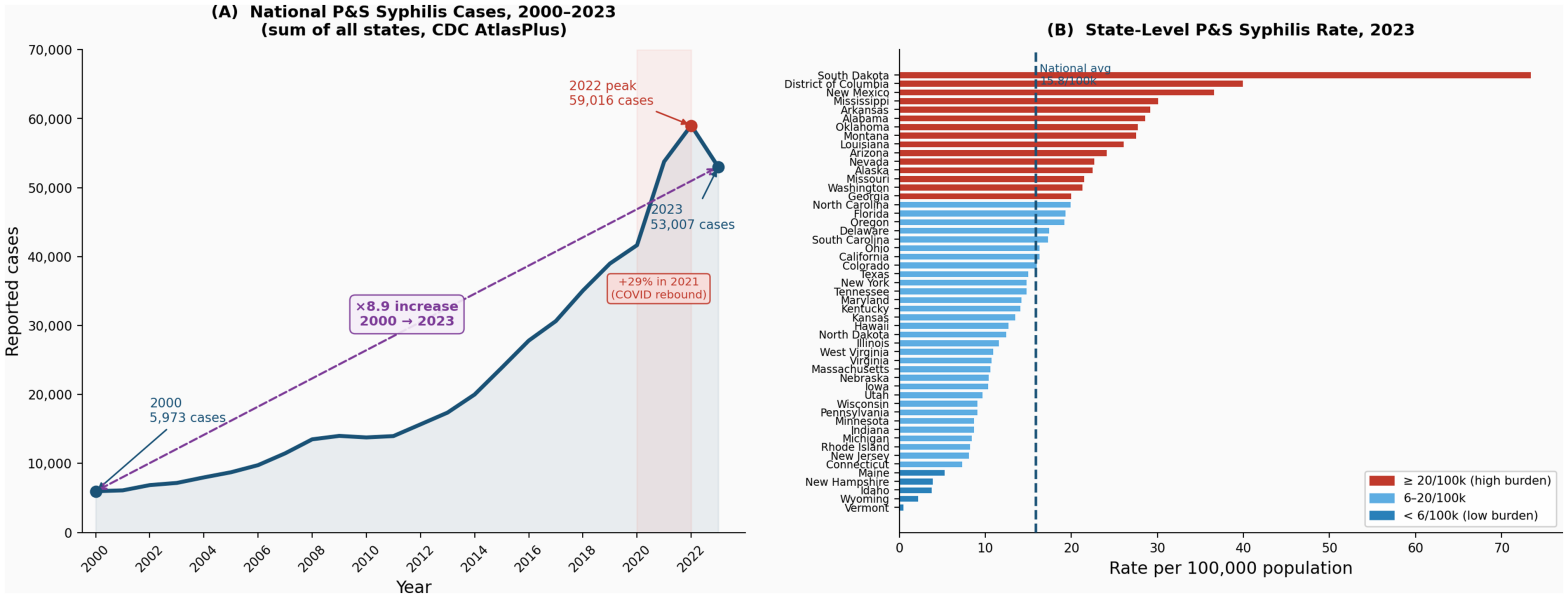
Epidemiological burden of primary and secondary syphilis in the United States, 2000–2023. **(A)** National reported primary and secondary syphilis cases from 2000 to 2023. **(B)** State-level primary and secondary syphilis rates in 2023.

Primary and secondary (P&S) syphilis has re-emerged as a major public health threat in the United States following decades of decline. After reaching historically low levels in the early 2000s, reported P&S syphilis cases increased nearly 8.9-fold, from 5,973 cases in 2000 to a peak of 59,016 cases in 2022 (see Figure 1), representing the highest burden recorded in over 23 years (1). Although a modest decline was observed in 2023 (53,007 cases), rates remain at historically elevated levels, underscoring the persistence of this epidemic. The trajectory of this resurgence has not been linear: a particularly sharp acceleration occurred between 2020 and 2021, when reported cases increased by 29% in a single year, coinciding with the disruption of sexually transmitted infection (STI) screening and treatment services during the coronavirus disease 2019 (COVID-19) pandemic (1).

The national aggregate figures mask profound geographic disparities in the distribution of P&S syphilis across the United States. In 2023, state-level rates ranged from 0.5 per 100,000 population in Vermont to 73.4 per 100,000 in South Dakota—a 147-fold difference (see Figure 1). High-burden states were concentrated in the South and certain Western states, including Mississippi (30.1/100,000), Arkansas (29.2/100,000), Alabama (28.6/100,000), and New Mexico (36.6/100,000), while Northeastern states generally maintained lower rates. Consistent with this geographic heterogeneity, Chang et al. found that county-level P&S syphilis hot-spots were mainly concentrated in Southern states, with additional hot-spots identified in Maryland and California (2). This spatially uneven burden suggests that syphilis prevention and control should not rely on a uniform, one-size-fits-all national strategy. Instead, public health resources should be allocated according to geographically specific epidemic patterns. State-specific forecasts are therefore important for anticipating future epidemic trajectories, identifying jurisdictions at risk of increasing burden, and guiding the targeted allocation of surveillance, screening, and prevention resources.

Existing research on syphilis forecasting in the United States can be broadly grouped into several categories. First, a substantial proportion of studies have been regional in scope, focusing on selected local jurisdictions rather than providing nationwide forecasts (3). Although these studies offer valuable local insights, their utility for national-level prevention planning and resource allocation remains limited. Second, some nationwide forecasting efforts have appeared primarily as conference abstracts or preliminary reports, suggesting early interest in national- or state-level prediction but offering limited methodological detail and no comprehensive estimates of the future burden (4). Third, existing nationwide predictive modeling work has largely focused on evaluating the predictive value of Google search engine data for syphilis surveillance rather than generating long-term, state-specific forecasts of the future case counts (5). Moreover, most prior studies have not incorporated socioeconomic and co-infection-related covariates, such as gonorrhea incidence, chlamydia rates, human immunodeficiency virus (HIV) prevalence, poverty, and unemployment, despite evidence that these factors are epidemiologically associated with syphilis transmission (6, 7). Probabilistic forecasts with formally calibrated uncertainty intervals have been largely absent, limiting the utility of existing projections for risk communication and resource planning.

To address these gaps, we develop a multi-model ensemble forecasting framework that integrates machine learning, neural networks, and statistical models at the state level, leveraging both autoregressive features and a broad set of socioeconomic and co-infection covariates, to generate calibrated 10-year forecasts of P&S syphilis incidence across all 50 US states and the District of Columbia.

## 2 Materials and methods

**Figure 2:**
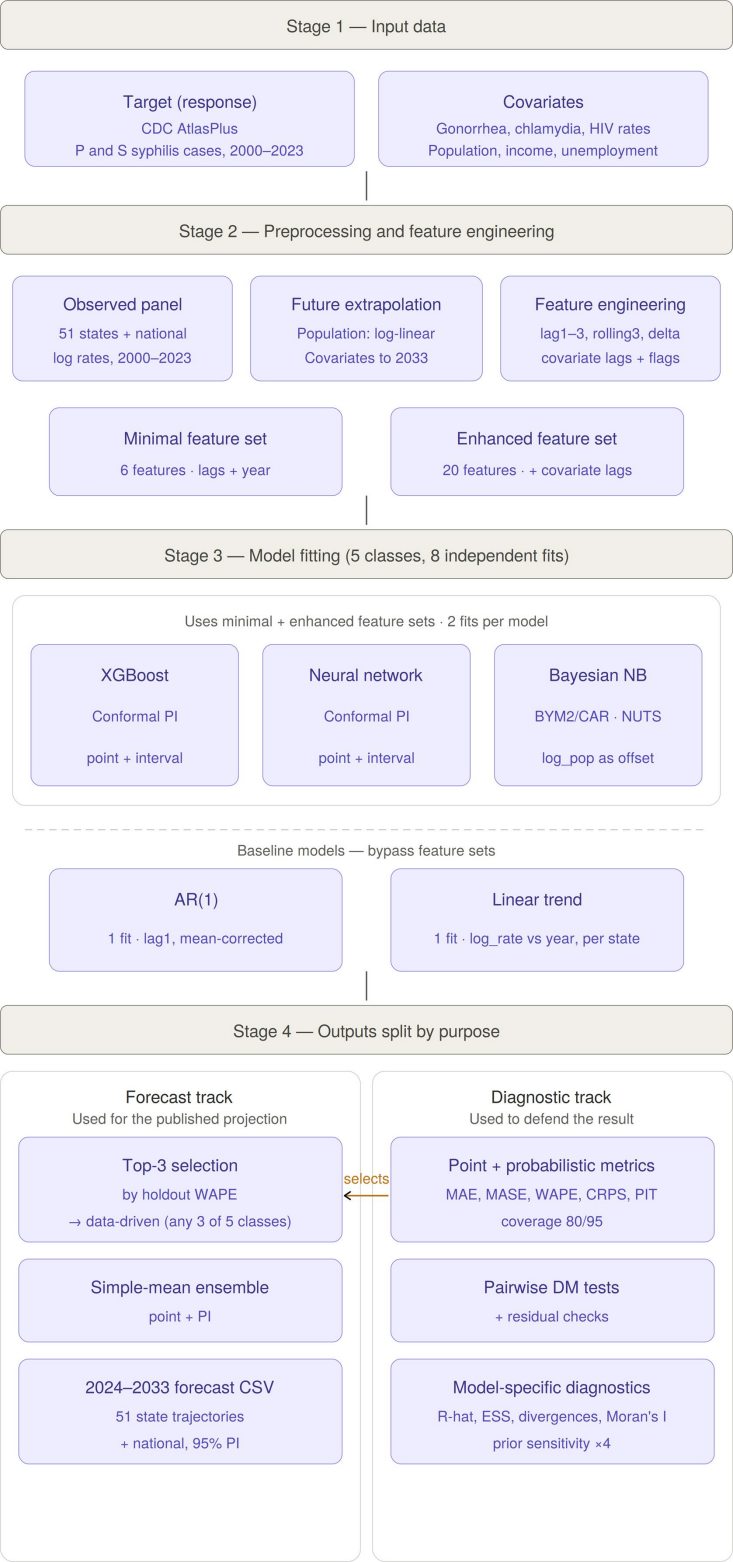
Workflow of the state-level ensemble forecasting framework for primary and secondary syphilis in the United States.

Additional implementation details on preprocessing, feature construction, future covariate extrapolation, interval calibration, national aggregation, model parameters, and prior specifications are provided in Supplementary Text S1. Additional details on predictor timing, temporal information boundaries, observed vs. extrapolated inputs and leakage audit are provided in Supplementary Text S3. Figure 2 provides an overview of the study workflow and is intended to help readers follow the data-processing and modeling procedures described in the following sections.

### 2.1 Outcome variable

The outcome was the annual number of reported primary and secondary (P&S) syphilis cases for each U. S. state and the District of Columbia from 2003 to 2023. Data were obtained from the Centers for Disease Control and Prevention (CDC) AtlasPlus surveillance tables based on the National Notifiable Diseases Surveillance System (NNDSS) reporting.

### 2.2 Covariates

Covariates included autoregressive features derived from prior syphilis burden, including lagged log-rate, recent rolling mean log-rate, and annual change; sexually transmitted infection covariates, including gonorrhea, chlamydia, and HIV log-rates (with HIV missingness indicators); socioeconomic variables, including poverty rate, unemployment rate, and median household income; and population data. Population was used as an offset in the Bayesian count model and for converting predicted rates to case counts in the other models.

### 2.3 Study design

**Figure 3:**
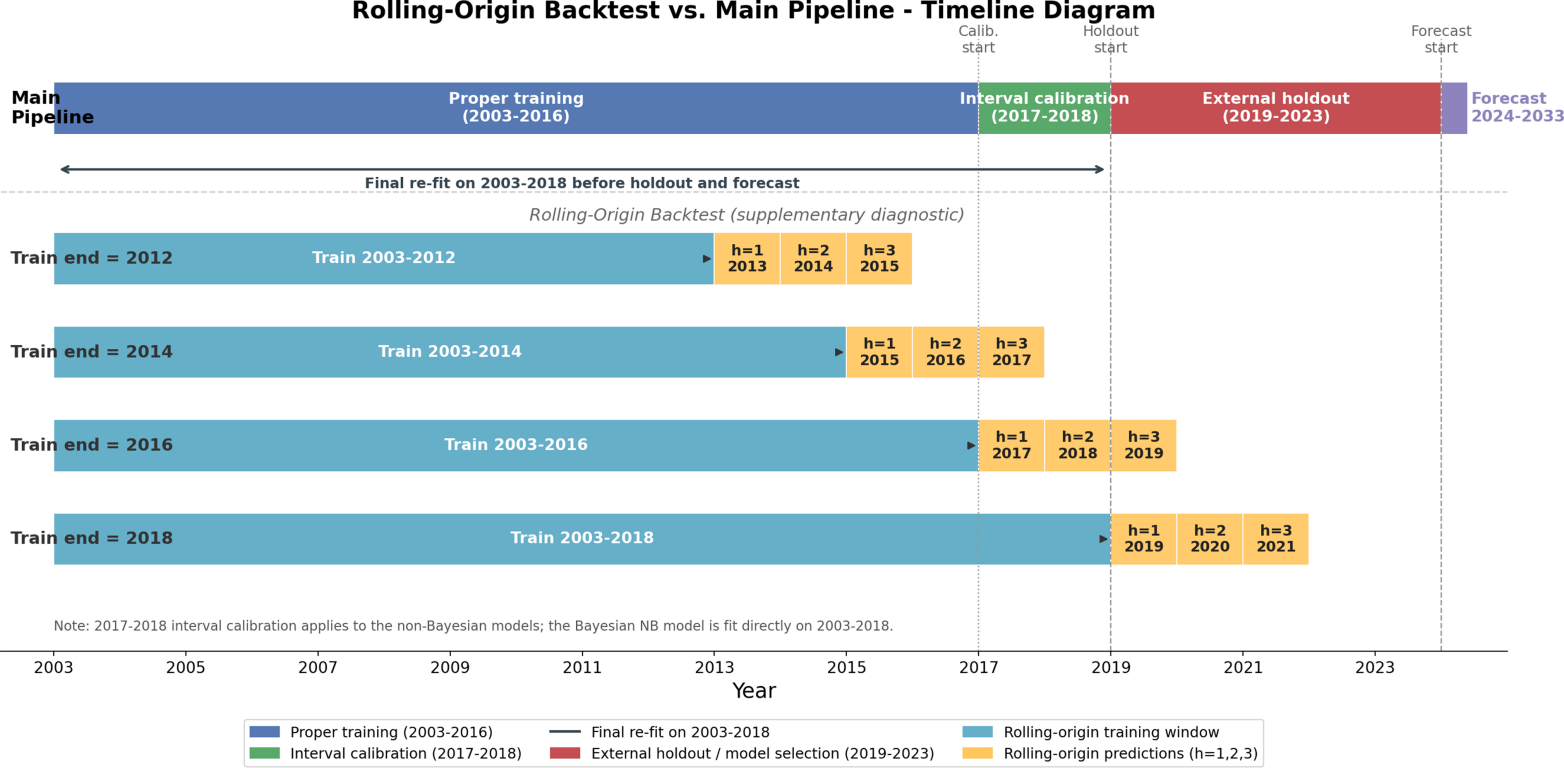
Timeline of the main forecasting pipeline and rolling-origin backtesting.

The study used a clearly defined temporal split. Data from 2000–2002 were used to construct lagged features, whereas the overall fitting window was 2003–2018. For the non-Bayesian models, 2003–2016 served as the internal training period, and 2017–2018 served as the internal residual-based interval-calibration period. Prediction intervals for the non-Bayesian models were calibrated using empirical residuals from the 2017–2018 calibration period, and 200 predictive samples were retained per observation, from which the 10th/90th and 2.5th/97.5th percentile intervals were derived (we conducted a sensitivity analysis on the length of the calibration window, and the detailed results are presented in Supplementary Text S2). After this internal development stage, the non-Bayesian models were re-fit on the full pre-holdout window (2003–2018) and then evaluated on the external holdout period from 2019–2023. The Bayesian model was fit directly on the 2003–2018 fitting window, and Bayesian uncertainty intervals were obtained from the posterior predictive distribution. Future forecasting covered 2024–2033 (see Figure 3).

We evaluated five model families: a state-specific linear trend baseline, a first-order autoregressive model of order 1 [AR(1)], Extreme Gradient Boosting (XGBoost), a Bayesian negative binomial model, and a neural network. For the covariate-dependent models, namely XGBoost, Bayesian negative binomial, and neural network, we considered two feature settings (minimal and enhanced). In contrast, the state-specific linear trend baseline and AR(1) were included as baseline comparators and were not substantively differentiated by feature setting. Model comparison was conducted only on the state-level external holdout set, with weighted absolute percentage error (WAPE) as the primary selection criterion, and symmetric mean absolute percentage error (sMAPE), and mean absolute scaled error (MASE) as secondary performance measures; the national aggregate was not used for model selection.

**Figure 4:**
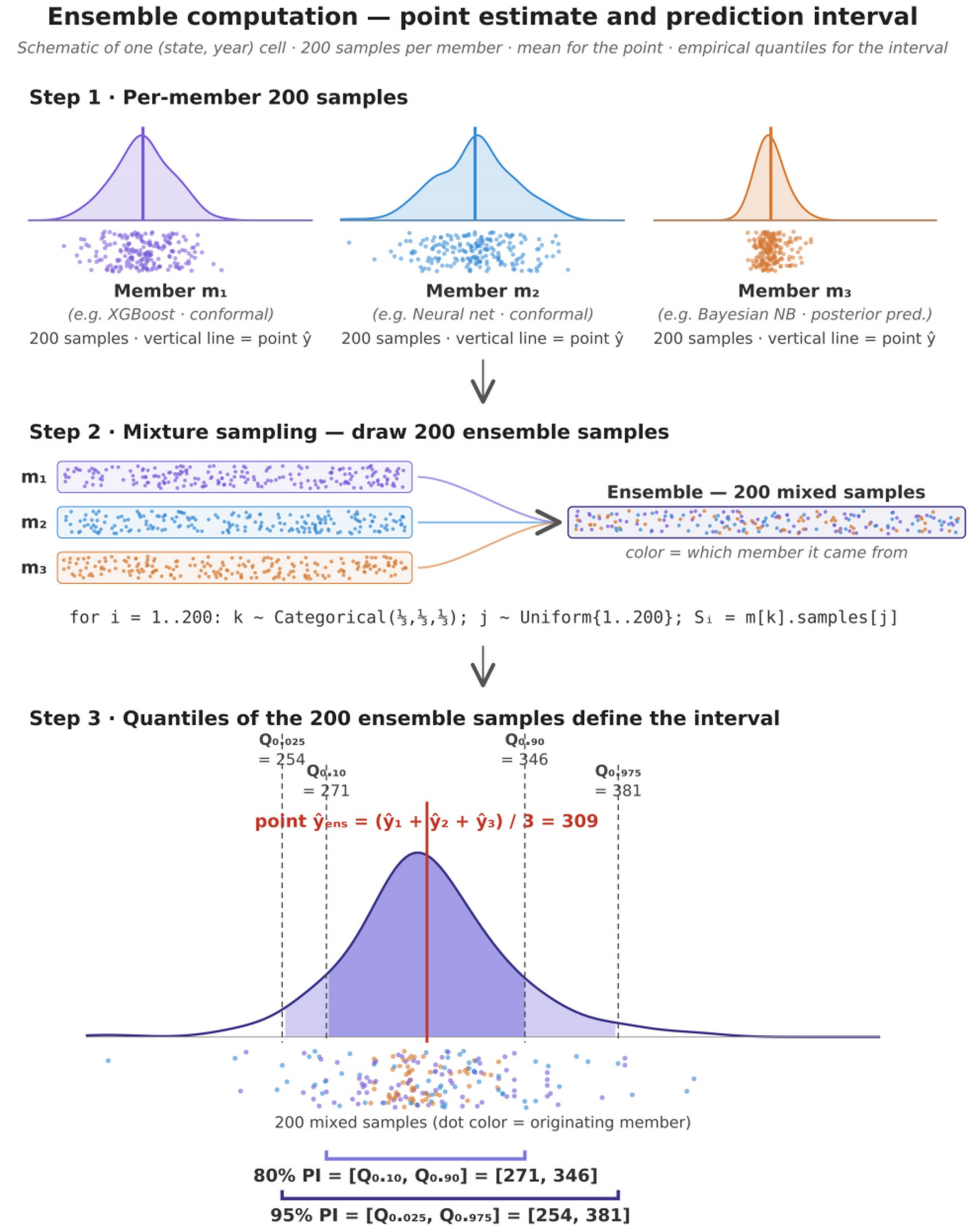
Ensemble computation for point forecasts and prediction intervals.

In the final run, the three best-performing models were enhanced XGBoost, enhanced neural network, and the state-specific linear trend baseline. At the state level, the final ensemble point forecast was defined as the equal-weight mean of the three-member point forecasts. State-level predictive intervals and probabilistic summaries were derived from equally weighted mixture sampling of the member predictive sample sets, with the 80 and 95% prediction intervals defined by the 10th/90th and 2.5th/97.5th percentiles, respectively. National forecasts were then obtained by summing the state-level ensemble predictive samples within each year and applying the median- and percentile-based summaries to the resulting national predictive distribution (see Figure 4).

### 2.4 Candidate models

The candidate models were selected to represent complementary forecasting paradigms rather than to benchmark all possible algorithms exhaustively. The state-specific linear trend and AR(1) models provided transparent statistical baselines; XGBoost represented a gradient-boosted tree approach capable of modeling non-linear covariate relationships; the neural network represented a flexible non-linear function approximator; the Bayesian negative binomial (Besag–York–Mollié 2 model/conditional autoregressive model [BYM2/CAR]) model represented a count-data framework with posterior uncertainty and spatial structure; and the final ensemble was used to reduce reliance on any single model family. We selected XGBoost as the representative tree-boosting method rather than adding multiple closely related boosting variants, because the primary aim of this study was to develop a robust state-level forecasting framework for estimating future P&S syphilis burden, rather than to conduct an exhaustive algorithmic benchmark. Although additional algorithms could be explored in future work, the present model set was intended to provide a balanced and interpretable forecasting framework across statistical, machine-learning, neural-network, Bayesian spatial count, and ensemble approaches. Detailed parameters and prior specifications are provided in Supplementary Text S1.

For state i in year t , let $C_{\mathrm{it}}$ denote the annual number of reported P&S syphilis cases, $P_{\mathrm{it}}$ denote the population size, $r_{\mathrm{it}}=100,000\times C_{\mathrm{it}}/P_{\mathrm{it}}$ denote the annual rate per 100,000 population, and $y_{\mathrm{it}}=\log(1+r_{\mathrm{it}})$ denote the log-transformed syphilis rate.

For models estimated on the log-rate scale, predicted case counts were obtained by back-transformation,

$${\hat{C}}_{\mathrm{it}}=P_{\mathrm{it}}(\exp({\hat{y}}_{\mathrm{it}})-1)/100,000.$$

The minimal non-Bayesian feature set primarily included the calendar-year index, the previous-year syphilis log-rate $\left(y_{i,t-1}\right)$ , the recent 3-year rolling mean log-rate, the previous-year change in log-rate, and $\log(P_{\mathrm{it}})$ .

The enhanced feature set further incorporated lagged and rolling gonorrhea and chlamydia log-rates, lagged poverty rate, unemployment rate, and log median household income, their recent changes, and HIV-related predictors, including missingness indicators.

A national-reference indicator was retained in the processed/reference panel; however, national records were excluded from model fitting, tuning, and evaluation, so all primary model estimation was performed on state-level observations only.

#### 2.4.1 State-specific linear trend baseline

The state-specific linear trend baseline was specified separately for each state as a linear time-trend model on the log-rate scale (8):

$$y_{\mathrm{it}}=\alpha_{i}+\beta_{i}t+\varepsilon_{\mathrm{it}},$$

where $y_{\mathrm{it}}$ is the log-transformed syphilis rate for the state i in year t , where t denotes calendar time, $\alpha_{i}$ is the state-specific intercept, $\beta_{i}$ is the state-specific linear time-trend coefficient, and $\varepsilon_{\mathrm{it}}$ is the residual term.

This model extrapolates future burden solely from the historical linear trend in syphilis log-rates and does not incorporate additional covariates.

In the current pipeline, this model was treated as a single-variant baseline comparator rather than as substantively distinct minimal and enhanced variants.

#### 2.4.2 AR(1)

The AR(1) baseline model was specified in mean-corrected form as (9):

$$y_{\mathrm{it}}-\mu_{i}=\phi_{i}(y_{i,t-1}-\mu_{i})+\varepsilon_{\mathrm{it}},|\phi_{i}|<1,$$

where $\mu_{i}$ is the mean training-period log-rate for state i , $\phi_{i}$ is the first-order autoregressive coefficient, and $\varepsilon_{\mathrm{it}}$ is the residual term. The stationarity constraint $|\phi_{i}|<1$ was imposed to prevent long-horizon recursive divergence. As with the naive drift model, the AR(1) model was handled as a single baseline specification and did not differ substantively between the minimal and enhanced branches of the pipeline.

#### 2.4.3 XGBoost

##### 2.4.3.1 Minimal model

The minimal XGBoost model predicted the syphilis log-rate as (10)

$${\hat{y}}_{\mathrm{it}}=f_{\mathrm{XGB}}\;(x_{\mathrm{it}}^{\left(\min\right)},z_{i})=\sum_{m=1}^{M}f_{m}\;(x_{\mathrm{it}}^{\left(\min\right)},z_{i}),$$

where $x_{\mathrm{it}}^{\left(\min\right)}$ is the minimal numeric feature vector for state i in year t , $z_{i}$ is the one-hot-encoded state identifier, $f_{m}(\cdot )$ is the m th regression tree, and M is the total number of trees in the boosted ensemble. In the minimal specification, $x_{\mathrm{it}}^{\left(\min\right)}$ included the year index, the previous-year syphilis log-rate, the recent 3-year rolling mean log-rate, the previous-year change in syphilis log-rate, and log-population. The inclusion of $z_{i}$ allowed the model to capture state-specific baseline differences.

##### 2.4.3.2 Enhanced model

The enhanced XGBoost model retained the same functional form,

$${\hat{y}}_{\mathrm{it}}=f_{\mathrm{XGB}}\;(x_{\mathrm{it}}^{\left(\mathrm{enh}\right)},z_{i})=\sum_{m=1}^{M}f_{m}\;(x_{\mathrm{it}}^{\left(\mathrm{enh}\right)},z_{i}),$$

but replaced the minimal numeric feature vector with an enhanced vector $x_{\mathrm{it}}^{\left(\mathrm{enh}\right)}$ . In addition to the minimal features, $x_{\mathrm{it}}^{\left(\mathrm{enh}\right)}$ included lagged and rolling gonorrhea log-rates, lagged and rolling chlamydia log-rates, lagged poverty rate, lagged unemployment rate, lagged log median household income, the corresponding annual changes in poverty, unemployment, and income, and lagged and rolling HIV log-rates together with HIV missingness indicators. The one-hot-encoded state identifier $z_{i}$ was retained in the enhanced model.

#### 2.4.4 Neural network (multilayer perceptron)

##### 2.4.4.1 Minimal model

The minimal neural network model was a two-hidden-layer feed-forward network (11, 12):

$$h_{\mathrm{it}}^{\left(1\right)}=g\;\;(W_{1}[x_{\mathrm{it}}^{\left(\min\right)},z_{i}]+b_{1}),$$

$$h_{\mathrm{it}}^{\left(2\right)}=g\;\;(W_{2}h_{\mathrm{it}}^{\left(1\right)}+b_{2}),$$

$${\hat{y}}_{\mathrm{it}}=W_{3}h_{\mathrm{it}}^{\left(2\right)}+b_{3},$$

where ${\hat{y}}_{\mathrm{it}}$ is the predicted log-transformed syphilis rate for state i in year t ; $x_{\mathrm{it}}^{\left(\min\right)}$ is the minimal numeric feature vector; $z_{i}$ is the one-hot-encoded state identifier; $\left[x_{\mathrm{it}}^{\min},z_{i}\right]$ denotes concatenation of the numeric and categorical inputs; $h_{\mathrm{it}}^{\left(1\right)}$ and $h_{\mathrm{it}}^{\left(2\right)}$ are the first and second hidden-layer representations; $W_{1}$ , $W_{2}$ , and $W_{3}$ are weight matrices; $b_{1}$ , $b_{2}$ , and $b_{3}$ are bias terms; and g(·) denotes the ReLU activation function. In the implemented architecture, the two hidden layers contained 96 and 48 neurons, respectively. The minimal numeric feature vector included the year index, the previous-year syphilis log-rate, the recent 3-year rolling mean log-rate, the previous-year change in log-rate, and log-population. State identifiers were also included as one-hot-encoded categorical features to enable the model to capture state-specific baseline differences. Dropout regularization was applied after each hidden layer.

##### 2.4.4.2 Enhanced model

The enhanced neural network retained the same architecture:

$$h_{\mathrm{it}}^{\left(1\right)}=g\;(W_{1}[x_{\mathrm{it}}^{\left(\mathrm{enh}\right)},z_{i}]+b_{1}),$$

$$h_{\mathrm{it}}^{\left(2\right)}=g\;(W_{2}h_{\mathrm{it}}^{\left(1\right)}+b_{2}),$$

$${\hat{y}}_{\mathrm{it}}=W_{3}h_{\mathrm{it}}^{\left(2\right)}+b_{3},$$

where ${\hat{y}}_{\mathrm{it}}$ , $z_{i}$ , $h_{\mathrm{it}}^{\left(1\right)}$ , $h_{\mathrm{it}}^{\left(2\right)}$ , $W_{1}$ , $W_{2}$ , $W_{3}$ , $b_{1}$ , $b_{2}$ , $b_{3}$ , and g(·) have the same interpretation as in the minimal model, while $x_{\mathrm{it}}^{\left(\mathrm{enh}\right)}$ denotes the enhanced numeric feature vector. Compared with the minimal specification, $x_{\mathrm{it}}^{\left(\mathrm{enh}\right)}$ additionally incorporated STI, socioeconomic, and HIV-related predictors. The one-hot-encoded state identifier $z_{i}$ and dropout regularization was retained in the enhanced model.

#### 2.4.5 Bayesian negative binomial (BYM2/CAR)

##### 2.4.5.1 Minimal model

The minimal Bayesian count model was specified as

$$C_{\mathrm{it}}|\mu_{\mathrm{it}},\alpha ∼\text{NegBin}(\mu_{\mathrm{it}},\alpha ),$$

$$\log(\mu_{\mathrm{it}})=\log(P_{\mathrm{it}}/100,000)+\beta_{0}+{\left(x_{\mathrm{it}}^{\left(\min,B\right)}\right)}^{T}\beta +u_{i},$$

where $C_{\mathrm{it}}$ is the observed annual case count, $\mu_{\mathrm{it}}$ is the expected annual case count, α is the overdispersion parameter (13), $\beta_{0}$ is the intercept, ${\left(x_{\mathrm{it}}^{\left(\min,B\right)}\right)}^{T}\beta$ denotes the linear predictor formed by the minimal Bayesian feature vector and its corresponding regression coefficients, and $u_{i}$ is the state-level random effect. The term $\log(P_{\mathrm{it}}/100,000)$ is a population offset (14), so log-population was not entered as an ordinary predictor in the Bayesian model.

The state-level random effect followed a Besag–York–Mollié 2 model (BYM2)/conditional autoregressive model (CAR)-type decomposition (15):

$$u_{i}=\sigma_{u}[\sqrt{1-\rho }\;v_{i}+\sqrt{\rho }\;s_{i}],$$

where $\sigma_{u}$ is the overall scale of the state effect, ρ controls the proportion of spatially structured variation, $v_{i}$ is the unstructured state-specific random effect, and $s_{i}$ is the structured spatial effect defined through the state adjacency matrix.

In the Bayesian formulation, prior distributions were assigned to the unknown parameters, and inference was based on the posterior distribution

$$p(\theta |C)\propto [\prod_{i,t}\text{NegBin}(C_{\mathrm{it}}|\mu_{\mathrm{it}},\alpha )]p(\theta ),$$

where θ denotes the collection of unknown model parameters and random effects, and p(θ) denotes their joint prior distribution. Forecasts and uncertainty intervals were then obtained from the posterior predictive distribution.

The minimal Bayesian feature vector $x_{\mathrm{it}}^{\left(\min,B\right)}$ included the year index, the previous-year syphilis log-rate, the recent 3-year rolling mean log-rate, the previous-year change in log-rate, and the national indicator, with log-population omitted because it was included through the offset.

##### 2.4.5.2 Enhanced model

The enhanced Bayesian model retained the same negative binomial likelihood and Bayesian hierarchical structure:

$$C_{\mathrm{it}}|\mu_{\mathrm{it}},\alpha ∼\text{NegBin}(\mu_{\mathrm{it}},\alpha ),$$

$$\log(\mu_{\mathrm{it}})=\log(P_{\mathrm{it}}/100,000)+\beta_{0}+{\left(x_{\mathrm{it}}^{\left(\mathrm{enh},B\right)}\right)}^{T}\beta +u_{i},$$

with the same interpretation of $C_{\mathrm{it}}$ , $\mu_{\mathrm{it}}$ , α , $\beta_{0}$ , and $u_{i}$ , and the population offset. Here, ${\left(x_{\mathrm{it}}^{\left(\mathrm{enh},B\right)}\right)}^{T}\beta$ denotes the linear predictor formed by the enhanced Bayesian feature vector and its corresponding regression coefficients.

The enhanced Bayesian feature vector extended the minimal Bayesian feature vector by adding lagged and rolling gonorrhea and chlamydia log-rates, lagged socioeconomic indicators and their annual changes, and HIV-related predictors, including missingness indicators.

The same BYM2/CAR spatial random-effect structure was used:

$$u_{i}=\sigma_{u}[\sqrt{1-\rho }\;v_{i}+\sqrt{\rho }\;s_{i}].$$

As in the minimal Bayesian model, prior distributions were assigned to the unknown parameters, posterior inference combined the negative binomial likelihood with these priors, and forecasts with uncertainty intervals were derived from the posterior predictive distribution.

Full details of the model parameterization and prior specification are provided in Supplementary Text S1.

### 2.5 Model evaluation and diagnostics

Model performance was evaluated from both point-prediction and probabilistic perspectives. For point forecasts, we summarized predictive accuracy using weighted absolute percentage error (WAPE), mean absolute error (MAE), root mean squared error (RMSE), sMAPE, mean absolute scaled error (MASE), and mean bias. Probabilistic forecast performance was assessed using continuous ranked probability score (CRPS), empirical coverage of the 80 and 95% prediction intervals, and probability integral transform (PIT)-based calibration diagnostics. Differences in predictive accuracy between competing models were further examined using the Diebold-Mariano test.

Additional diagnostics were used to assess model adequacy and robustness. Residual-based diagnostics were examined to identify a systematic lack of fit. As a supplementary robustness analysis, rolling-origin backtesting was performed using expanding training windows with endpoints in 2012, 2014, 2016, and 2018. At each origin, models were re-fit using all available data from 2003 through the origin year and then recursively projected 1-, 2-, and 3-year ahead forecasts. Horizon-specific predictive performance was summarized primarily by weighted absolute percentage error (WAPE). In the standard run, rolling-origin backtesting was applied to the non-Bayesian models, whereas the Bayesian negative binomial model was not included because repeated posterior sampling across multiple rolling training windows was computationally intensive. For the Bayesian model, forecast uncertainty was quantified using posterior predictive distributions obtained from posterior sampling. Robustness was further examined through prior sensitivity analyses under three alternative prior specifications.

To assess whether important spatial structure remained unexplained after model fitting, we also examined Moran’s I for state-level residuals using a Queen contiguity weight matrix. The purpose of this analysis was not to treat spatial autocorrelation as a primary study endpoint, but rather to evaluate whether geographically patterned residual dependence persisted, suggesting incomplete capture of spatially structured variation relevant to state-level forecasting.

## 3 Results

### 3.1 Descriptive statistics

**Figure 5:**
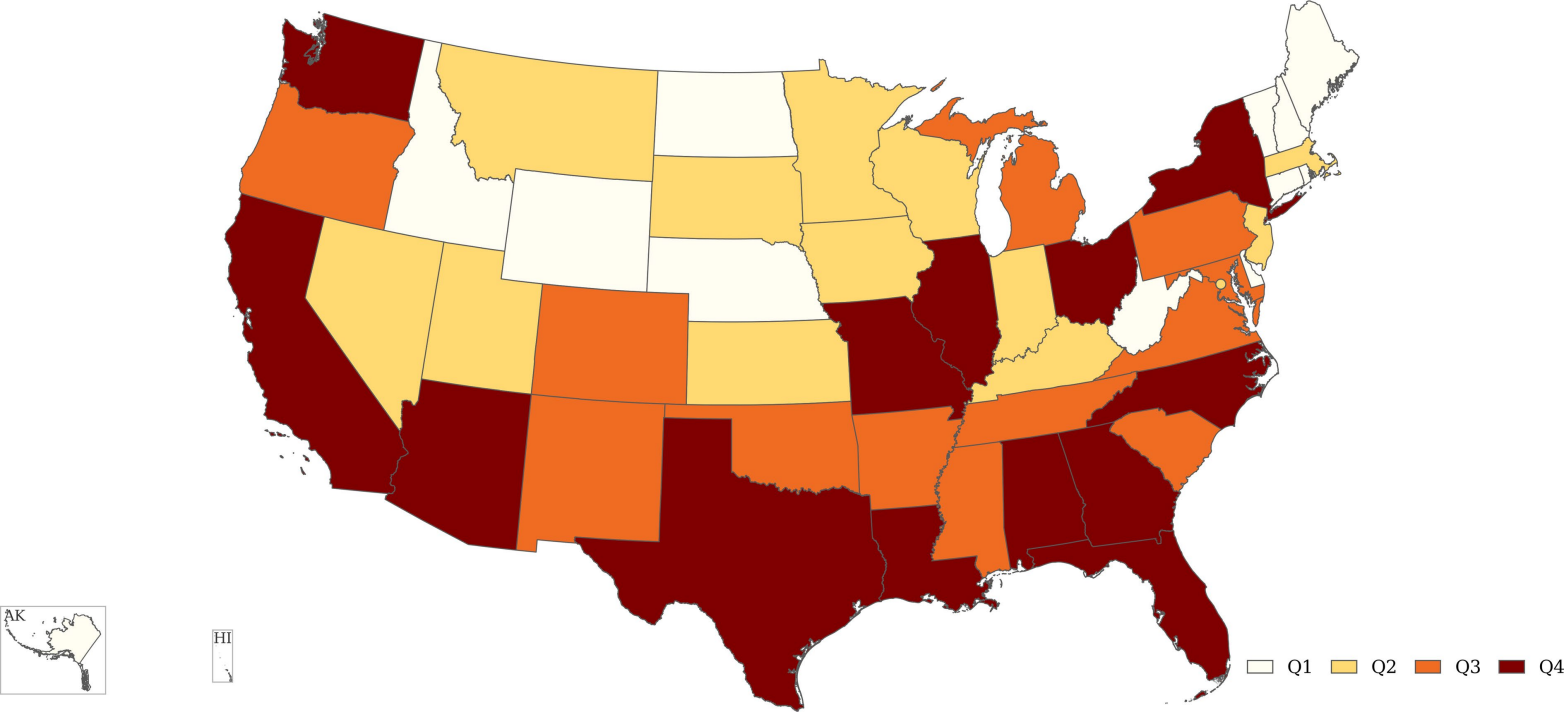
Quartile distribution of 2023 state-level primary and secondary syphilis case counts in the United States.

For descriptive purposes, jurisdictions were first grouped into empirical quartiles according to their 2023 P&S syphilis case counts. The observed ranges were 3–265 cases in the lowest quartile ( n=13 ), 271–751 in the second quartile ( n=13 ), 773–1,179 in the third quartile ( n=12 ), and 1,196–6,348 in the highest quartile ( n=13 ). No state or jurisdiction had fewer cases in 2023 than in 2003. These findings indicate marked interstate heterogeneity in P&S syphilis burden over 2003–2023 despite a uniformly increasing trajectory across jurisdictions (see Figure 5).

Covariate completeness was generally high. In the state-level panel from 2003 to 2023, gonorrhea, chlamydia, poverty rate, unemployment rate, and median household income had no missing values; the human immunodeficiency virus (HIV) indicator had a missingness rate of 23.8%, mainly because available surveillance data began in 2008. Some correlations were observed among covariates, but no complete redundancy was identified: the highest correlation was between gonorrhea and chlamydia log-rates ( r=0.785 ); chlamydia and HIV log-rates showed a moderate positive correlation ( r=0.601 ); and poverty rate was moderately negatively correlated with log median household income ( r=−0.593 ).

### 3.2 Model diagnostics and predictive performance

For descriptive clarity, minimal and enhanced variants were treated as alternative specifications within the same model family rather than as fully independent model classes; all candidate specifications are shown in Table 1. Among the model families, the enhanced XGBoost and the enhanced neural network showed the strongest external holdout performance. Enhanced XGBoost achieved a WAPE of 19.98%, an MAE of 193.1 cases, and a mean CRPS of 137.45; its rolling-origin WAPE values were 14.03, 19.26, and 24.77% for 1-, 2-, and 3-year-ahead forecasts, respectively. Enhanced neural network ranked second, with a WAPE of 24.63%, an MAE of 238.1, and a mean CRPS of 183.45; the corresponding rolling-origin WAPE values were 11.62, 16.95, and 20.33%.

**Table 1:** External holdout and rolling-origin predictive performance of candidate forecasting model specifications.

Specification	Holdout WAPE	Holdout MAE	Holdout mean CRPS	Holdout MASE	Rolling WAPE h = 1	Rolling WAPE h = 2	Rolling WAPE h = 3
Enhanced XGBoost	19.98%	193.1	137.45	5.68	14.03%	19.26%	24.77%
Enhanced neural network	24.63%	238.1	183.45	6.18	11.62%	16.95%	20.33%
State-specific linear trend baseline	25.54%	246.8	192.74	7.33	16.87%	19.07%	23.91%
AR(1) baseline	38.27%	369.8	205.22	10.12	17.09%	29.25%	42.22%
Enhanced Bayesian negative binomial	65.60%	634.0	486.95	10.00	—	—	—
Minimal XGBoost	22.70%	219.4	159.40	6.39	14.29%	19.19%	26.38%
Minimal neural network	26.42%	255.3	198.43	6.08	11.44%	16.37%	21.14%
Minimal Bayesian negative binomial	74.31%	718.2	535.65	10.21	—	—	—
Final top-3 global ensemble	20.83%	201.3	149.03	5.99	12.23%	15.85%	21.63%

At the other end, Bayesian negative binomial and AR(1) performed worst overall. Minimal Bayesian negative binomial yielded a WAPE of 74.31%, an MAE of 718.2, and a mean CRPS of 535.65. The AR(1) baseline had a WAPE of 38.27%, an MAE of 369.8, and a mean CRPS of 205.22, and its rolling-origin WAPE increased sharply from 17.09–29.25 and 42.22% across 1-, 2-, and 3-year-ahead forecasts.

### 3.3 Pairwise Diebold-Mariano test results

Pairwise Diebold-Mariano comparisons were used as a supplementary assessment of pooled state-year forecast loss differentials. Because the evaluation data have a state-year panel structure, with possible within-state temporal dependence and cross-state dependence, these *p*-values should be interpreted cautiously as supportive rather than definitive inferential evidence.

Pairwise Diebold-Mariano tests showed a clearer performance hierarchy in the enhanced feature set than in the minimal feature set. In the enhanced set, XGBoost and neural network performed better overall than the AR(1) and Bayesian negative binomial models, whereas the state-specific linear trend baseline was intermediate. Specifically, XGBoost significantly outperformed AR(1), the state-specific linear trend baseline, and Bayesian negative binomial, but did not differ significantly from neural network. In the minimal set, XGBoost, neural network, and the state-specific linear trend baseline did not differ significantly from one another, whereas all three outperformed AR(1) and Bayesian negative binomial; in addition, AR(1) significantly outperformed Bayesian negative binomial. Full pairwise Diebold-Mariano results are summarized in Table 2.

**Table 2:** Pairwise Diebold-Mariano comparisons of candidate forecasting models on the external holdout set.

Feature set	Model 1	Model 2	Better model	*p*-value	Significant
Enhanced	AR(1) baseline	Bayesian negative binomial	AR(1) baseline	0.0498	Yes
Enhanced	AR(1) baseline	State-specific linear trend baseline	State-specific linear trend baseline	<0.001	Yes
Enhanced	AR(1) baseline	Neural network	Neural network	<0.001	Yes
Enhanced	AR(1) baseline	XGBoost	XGBoost	<0.001	Yes
Enhanced	Bayesian negative binomial	State-specific linear trend baseline	State-specific linear trend baseline	0.0022	Yes
Enhanced	Bayesian negative binomial	Neural network	Neural network	<0.001	Yes
Enhanced	Bayesian negative binomial	XGBoost	XGBoost	0.0014	Yes
Enhanced	State-specific linear trend baseline	Neural network	No significant difference	0.6433	No
Enhanced	State-specific linear trend baseline	XGBoost	XGBoost	0.0309	Yes
Enhanced	Neural network	XGBoost	No significant difference	0.1038	No
Minimal	AR(1) baseline	Bayesian negative binomial	AR(1) baseline	0.0059	Yes
Minimal	AR(1) baseline	State-specific linear trend baseline	State-specific linear trend baseline	<0.001	Yes
Minimal	AR(1) baseline	Neural network	Neural network	0.0036	Yes
Minimal	AR(1) baseline	XGBoost	XGBoost	<0.001	Yes
Minimal	Bayesian negative binomial	State-specific linear trend baseline	State-specific linear trend baseline	<0.001	Yes
Minimal	Bayesian negative binomial	Neural network	Neural network	<0.001	Yes
Minimal	Bayesian negative binomial	XGBoost	XGBoost	<0.001	Yes
Minimal	State-specific linear trend baseline	Neural network	No significant difference	0.7548	No
Minimal	State-specific linear trend baseline	XGBoost	No significant difference	0.2594	No
Minimal	Neural network	XGBoost	No significant difference	0.3016	No

### 3.4 Ensemble forecasting results

The final ensemble combined the three top-ranked models selected on external holdout performance: enhanced XGBoost, enhanced neural network, and the state-specific linear trend baseline. On the external holdout set, the ensemble achieved a WAPE of 20.83%, an MAE of 201.33 cases, and a mean CRPS of 149.03. Although the final ensemble did not surpass enhanced XGBoost, the single best individual specification (WAPE 19.98%; mean CRPS 137.45), its performance closely followed that of enhanced XGBoost.

The final top-3 global ensemble achieved the strongest interval calibration overall, with empirical coverage of 76.1% for the nominal 80% prediction interval and 91.0% for the nominal 95% prediction interval. Among its three component models, enhanced XGBoost showed the strongest interval coverage (70.2 and 89.0%), followed by the state-specific linear trend baseline (66.7 and 85.1%), whereas enhanced neural network showed the weakest coverage (53.3 and 80.8%). Thus, although the ensemble did not surpass enhanced XGBoost in point-forecast accuracy on the external holdout set (WAPE 20.83% vs. 19.98%), it provided slightly better calibrated prediction intervals (see Table 3).

**Table 3:** Empirical coverage of 80 and 95% prediction intervals on the external holdout set.

Model specification	80% PI empirical coverage	95% PI empirical coverage
Enhanced XGBoost	70.20%	89.02%
Enhanced neural network	53.33%	80.78%
Enhanced Bayesian negative binomial	58.82%	74.90%
Minimal XGBoost	61.18%	86.27%
Minimal neural network	61.96%	87.06%
Minimal Bayesian negative binomial	58.04%	72.94%
State-specific linear trend baseline	66.67%	85.10%
AR(1) baseline	53.33%	86.67%
Final top-3 global ensemble	76.08%	90.98%

### 3.5 Spatial diagnostics: Moran’s I

Residual Moran’s I results differed across model classes. Significant positive residual spatial autocorrelation was observed for the AR(1) baseline (I = 0.231, permutation *p* = 0.009/0.008) and the state-specific linear trend baseline (I = 0.142, *p* = 0.041/0.027), indicating remaining geographic clustering in their prediction errors. Among the XGBoost specifications, enhanced XGBoost also showed significant positive residual spatial autocorrelation (I = 0.143, *p* = 0.048), whereas minimal XGBoost showed a similar but non-significant pattern (I = 0.126, *p* = 0.063). By contrast, both Bayesian negative binomial specifications, both neural network specifications, and the final top-3 ensemble showed no significant residual spatial autocorrelation (ensemble: I = 0.050, *p* = 0.269). Overall, these findings suggest that residual geographic clustering persisted in the simpler autoregressive baselines and, to a lesser extent, in XGBoost, whereas the neural network, Bayesian, and final ensemble models did not show statistically significant residual spatial dependence (see Table 4).

**Table 4:** Residual spatial autocorrelation diagnostics based on Moran’s I across candidate forecasting models.

Model specification	Moran’s I	Expected I	Permutation *p*-value	Interpretation
Enhanced XGBoost	0.143			Significant positive residual spatial autocorrelation
Enhanced neural network	−0.027	−0.021	0.459	No evidence of residual spatial autocorrelation
Enhanced Bayesian negative binomial	−0.014	−0.021	−0.021	0.048
Minimal XGBoost	0.126	−0.021	0.063	Weak positive residual spatial autocorrelation; not statistically significant
Minimal neural network	−0.021	−0.021	0.444	No evidence of residual spatial autocorrelation
Minimal Bayesian negative binomial	0.016	−0.021	0.257	No evidence of residual spatial autocorrelation
State-specific linear trend baseline	0.142	−0.021	0.027/0.041	Significant positive residual spatial autocorrelation
AR(1) baseline	0.231	−0.021	0.008/0.009	Significant positive residual spatial autocorrelation
Final top-3 global ensemble	0.050	−0.021	0.269	No evidence of residual spatial autocorrelation

AR(1) and the state-specific linear trend baseline were treated as single baseline comparators. In the raw diagnostic output, each baseline appeared twice with identical Moran’s I values but slightly different permutation *p*-values; both p-values are therefore reported.

### 3.6 Rolling-origin backtesting results

Rolling-origin backtesting showed that the three models ultimately retained in the final ensemble remained comparatively stable across increasing forecast horizons. The enhanced neural network had the lowest WAPE at all three horizons, increasing from 11.62% at 1 year ahead to 16.95% at 2 years ahead and 20.33% at 3 years ahead. Enhanced XGBoost showed a similar pattern, with WAPE values of 14.03, 19.26, and 24.77%, respectively, whereas the state-specific linear trend baseline yielded 16.87, 19.07, and 23.91%. Detailed rolling-origin results for all candidate models are presented in Table 5.

**Table 5:** Rolling-origin backtesting results for candidate forecasting model specifications.

Model class	Specification	WAPE (h = 1)	WAPE (h = 2)	WAPE (h = 3)	Note
XGBoost	Enhanced	14.03%	19.26%	24.77%	Final ensemble member
Neural network	Enhanced	11.62%	16.95%	20.33%	Final ensemble member
State-specific linear trend baseline	Single	16.87%	19.07%	23.91%	Final ensemble member
AR(1) baseline	Single	17.09%	29.25%	42.22%	
XGBoost	Minimal	14.29%	19.19%	26.38%	
Neural network	Minimal	11.44%	16.37%	21.14%	

### 3.7 2024–2033 forecast results

#### 3.7.1 At the national level

Under the extrapolated covariate trajectories and continuation of the main historical patterns represented in the data, the final top-3 ensemble projected an increase in reported P&S syphilis cases over 2024–2033, from 62,594 cases in 2024 to 131,736 cases in 2033. The corresponding 95% prediction intervals were 53,837–79,384 in 2024 and 108,313–172,716 in 2033. Within this conditional projection, no trough or reversal was projected during the forecast period.

**Figure 6:**
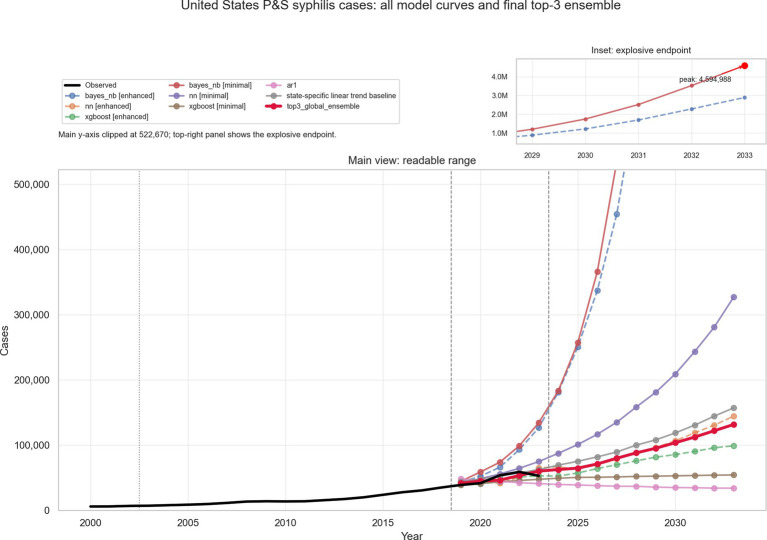
National primary and secondary syphilis forecasts from candidate models and the final top-3 ensemble for the United States, 2000–2033.

Figure 6 shows the observed national P&S syphilis cases and projected trajectories from all candidate models, highlighting the more moderate final top-3 ensemble forecast through 2033.

**Figure 7:**
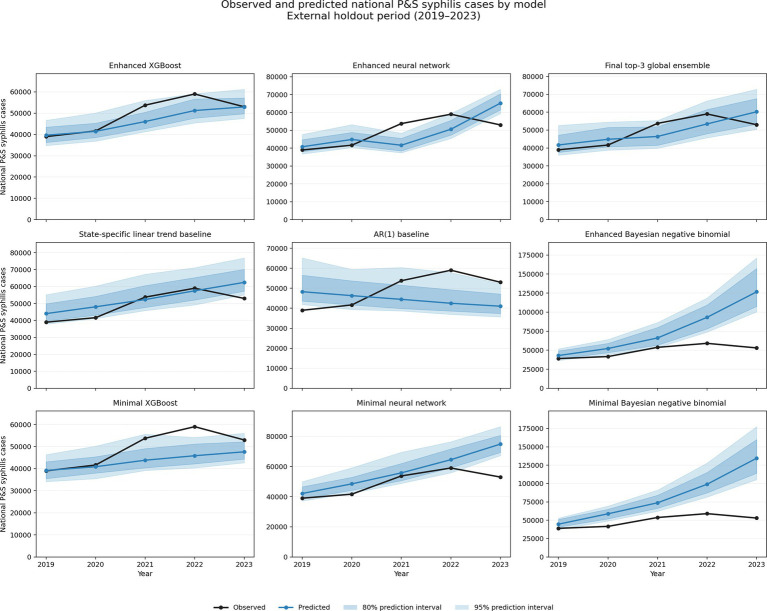
Further presents the external-holdout trajectories for each model separately, comparing observed national P&S syphilis cases with model-specific predicted values and prediction intervals over 2019–2023.

Figure 7 further presents the external-holdout trajectories for each model separately, comparing observed national P&S syphilis cases with model-specific predicted values and prediction intervals over 2019–2023.

#### 3.7.2 State-level results

State-level forecasts showed substantial heterogeneity in projected P&S syphilis burden across jurisdictions over 2024–2033. The largest cumulative projected burdens were concentrated in California (149,119.4 cases), Florida (72,310.7), New York (63,184.9), Texas (57,942.7), and Georgia (38,423.5)—together, these five states accounted for 45.7% of the total cumulative state-level predicted burden during the forecast period. Future growth was not confined to the highest-burden states. When growth was defined as the relative change from forecast 2024 to forecast 2033, the steepest increases were projected in Iowa (+264.0%), North Dakota (+244.0%), Wisconsin (+230.4%), South Dakota (+216.6%), and Nevada (+204.4%), indicating that several lower- or mid-burden jurisdictions may experience particularly rapid expansion over time. Prediction uncertainty was also unevenly distributed across jurisdictions. In terms of forecast uncertainty, measured as the relative width of the 95% prediction interval for the cumulative projected case total over 2024–2033, defined as (Q97.5–Q2.5) / median x 100%, the greatest uncertainty was observed in Nevada (104.4%), Oregon (101.7%), Vermont (83.8%), the District of Columbia (79.5%), and Wyoming (78.8%). Relative to observed 2023 case counts, only a few jurisdictions were projected to have a stable or lower burden by 2033, including South Dakota (−38.8%), Montana (−37.6%), New Mexico (−11.9%), and Alaska (−5.0%), suggesting that elevated P&S syphilis burden is likely to persist across most of the United States. Detailed state-level forecast estimates for each prediction year, including prediction intervals, are provided in Supplementary Table S1.

**Figure 8:**
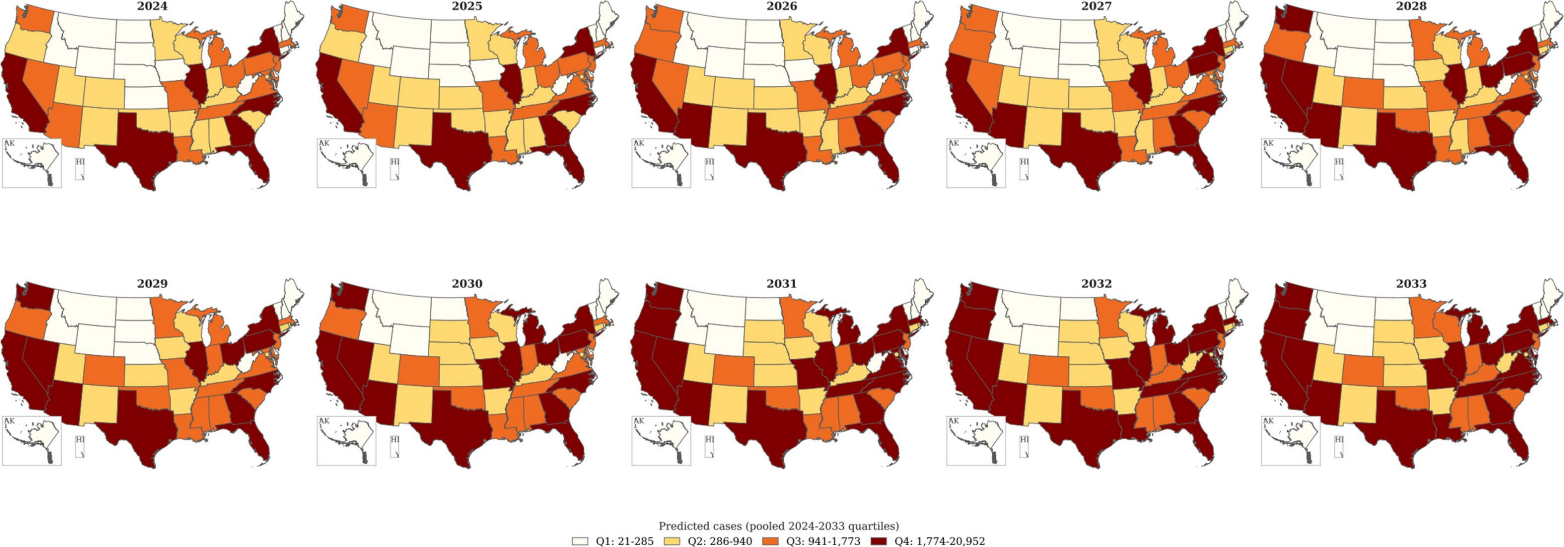
Annual state-level maps of forecasted primary and secondary syphilis cases in the United States, 2024–2033.

State-level forecasted case counts for 2024–2033 were mapped across the United States to illustrate geographic patterns and temporal changes in projected burden (see Figure 8).

## 4 Discussion

### 4.1 Principal findings

This study provides, to our knowledge, one of the first state-level forecasts of reported primary and secondary (P&S) syphilis for all 50 US states and the District of Columbia over 2024–2033. Several findings stand out. First, the forecasts indicate that national P&S syphilis burden is likely to remain high over the next decade, increasing from 62,594 projected cases in 2024 to 131,736 in 2033, with no projected trough or reversal during the forecast horizon. Second, candidate models differed substantially in performance, indicating that model choice materially affected both point forecasts and uncertainty estimates. Third, enhanced XGBoost and enhanced neural network were the two strongest individual specifications, whereas the final top-3 ensemble achieved near-best performance while reducing reliance on any single model family. Finally, projected burden remained highly heterogeneous across states, both in absolute case counts and in growth trajectories, suggesting that future national burden will continue to be shaped by a combination of persistent hot-spots and emerging growth areas.

### 4.2 Main contribution

The main contribution of this study is not the development of a new forecasting algorithm, but the application and evaluation of established forecasting methods to address an unresolved gap in applied public-health forecasting. Specifically, this study makes three contributions. First, it provides 10-year conditional forecasts of P&S syphilis burden for all 50 US states and the District of Columbia, while incorporating syphilis history, STI-related covariates, socioeconomic indicators, and population structure. Second, by comparing statistical, Bayesian, neural-network, machine-learning, and ensemble approaches under a common external-holdout and rolling-origin evaluation framework, it provides empirical evidence and a practical reference for model selection in similar subnational infectious-disease forecasting studies. Third, it translates state-level forecasts into public health-relevant categories by distinguishing jurisdictions with persistently high projected burden from those with rapid projected growth, thereby supporting interpretation for preparedness planning and resource allocation.

### 4.3 Robustness of the ensemble forecasting framework

The final ensemble, which combined enhanced XGBoost, enhanced neural network, and the state-specific linear trend baseline, showed strong state-level out-of-sample point-prediction performance on the external holdout set, with a WAPE of 20.83%, an MAE of 201.3 cases, and a mean CRPS of 149.03.

The final ensemble was supported by multiple validation diagnostics rather than a single accuracy cutoff. On the external holdout set, it achieved a WAPE of 20.83% and an sMAPE of 30.97% (compared with 25.54 and 40.80%, respectively, for the state-specific linear trend baseline). The mean CRPS was 149.03, and there was no evidence of residual spatial autocorrelation (Moran’s I = 0.050, permutation *p* = 0.269), suggesting no statistically significant spatial clustering in residual prediction errors across states. Together, these results suggest that the ensemble may provide a practical framework for state-level point forecasting. However, empirical interval coverage remained slightly below nominal levels (76.1% for nominal 80% intervals and 91.0% for nominal 95% intervals), so prediction intervals should be interpreted as approximate rather than perfectly calibrated (see Supplementary Table S2). However, a non-significant Moran’s I should not be interpreted as evidence that all spatial dependence has been fully captured, but rather as indicating that no statistically significant global spatial autocorrelation was detected in the evaluated residuals.

Although enhanced XGBoost achieved the best point-prediction performance on the fixed external holdout set, rolling-origin backtesting suggested that this advantage was not uniform across forecast horizons, with the enhanced neural network showing lower WAPE than enhanced XGBoost at all three evaluated horizons (see Table 1). This finding is important because rolling-origin evaluation has been recommended as a way to improve the efficiency and reliability of out-of-sample forecasting tests by using multiple forecast origins, recalibrating model coefficients, and evaluating performance across multiple test periods (16). This shift in model ranking suggests that no single model was uniformly dominant across evaluation settings. In addition, the final ensemble had lower rolling-origin WAPE than enhanced XGBoost at all three evaluated horizons. This result further supports retaining the final ensemble as the main forecasting model, because its advantage was more apparent in the rolling-origin robustness assessment than in the fixed external holdout evaluation.

In addition, residual Moran’s I for the final ensemble was close to zero and non-significant ( I=0.050 , permutation p=0.269 ), suggesting no evidence of residual geographic clustering in prediction errors on the contiguous-state diagnostic map. The three retained ensemble members also remained comparatively stable in rolling-origin backtesting, supporting their robustness across increasing forecast horizons.

National forecasts were not generated from a separately fitted national model. Instead, after the final ensemble was constructed at the state level, national predictions were obtained by summing the state-level ensemble ensemble predictions for each year. The resulting national projections therefore inherit the state-level ensemble structure and were evaluated only as aggregated state-sum forecasts. Treating the national series as an additional “state” in model fitting would place an aggregate time series of a different scale into a framework designed for state-level comparison and residual diagnostics, making those diagnostics less directly interpretable for the state-level forecasting objective. By instead deriving national forecasts as the sum of state-level ensemble predictive samples, we ensured that the reported national forecasts were numerically consistent with the corresponding state-level forecasts.

The poor performance of the Bayesian negative binomial model warrants specific comment. Its inflated long-horizon forecasts were likely driven not by the Bayesian framework itself but by the combination of strong upward lag signals in the training data and recursive multi-step forecasting, which repeatedly fed those signals back into future predictions, amplifying growth over time. Further evidence-based diagnostic details on the Bayesian model’s poor performance are provided in Supplementary Text S4.

The Bayesian negative binomial specification was retained despite its weaker holdout performance because it represented a methodologically distinct and epidemiologically well-motivated paradigm—a model-based hierarchical framework with CAR/BYM2 spatial random effects and a count-data likelihood suited to overdispersed, spatially dependent counts. Its inclusion served a deliberate methodological purpose: where there is limited *a priori* basis for selecting a single model class, evaluating a broad methodological spectrum under a unified protocol allows the data, rather than analyst preference, to inform model-class comparisons. Anchored by the Bayesian specification, our candidate set therefore spanned three paradigms—hierarchical Bayesian inference, classical parametric time-series baselines (AR1, state-specific linear trend baseline), and machine-learning forecasters (gradient boosting, neural networks)—all trained and evaluated under identical protocols. Within this candidate set, the Bayesian specification, as configured here, did not outperform the machine-learning models or the simple-average ensemble on our external holdout set. While a single application cannot generalize to the broader class of hierarchical Bayesian count-regression models, the pattern we observed is consistent with the possibility that strongly parametric likelihoods may be more susceptible than flexible function approximators to error accumulation under recursive multi-step propagation. The relative model-class ranking observed here may therefore offer a useful empirical reference for future infectious-disease forecasting studies in comparable long-horizon, sub-national settings, while leaving open the question of how these comparisons would translate to other diseases, geographies, and forecast horizons.

### 4.4 Implications of model comparison

The model comparison results suggest that more flexible approaches, particularly enhanced XGBoost, captured state-level P&S syphilis dynamics better than simpler traditional time-series baselines. Enhanced XGBoost significantly outperformed the AR(1) baseline (*p* < 0.001) and the state-specific linear trend baseline (*p* = 0.031), whereas its difference from the enhanced neural network was not statistically significant (*p* = 0.104). This pattern suggests that non-linear models may better accommodate heterogeneous state trajectories, changing epidemic intensity, and interactions among lagged epidemiologic and contextual signals. By contrast, AR(1) was clearly less reliable for long-horizon extrapolation: its rolling-origin WAPE increased from 17.09% at 1 year ahead to 29.25% at 2 years ahead and 42.22% at 3 years ahead, and its residual Moran’s I remained significantly positive. Together, these findings suggest that a simple stationary AR(1) model may be insufficient for forecasting a rapidly evolving and spatially heterogeneous epidemic over long horizons.

To assess the incremental value of additional covariate information, we specified both minimal and enhanced versions of the covariate-dependent models and used this contrast to examine whether adding STI-, socioeconomic-, and HIV-related predictors improved both point forecasting and interval forecasting. In the final results, the enhanced specification was associated with lower holdout WAPE for all three model families, improving from 22.70 to 19.98% for XGBoost, from 26.42 to 24.63% for the neural network, and from 74.31 to 65.60% for the Bayesian negative binomial model. The effect on empirical interval coverage was more mixed. For XGBoost, coverage improved from 61.18–86.27 to 70.20–89.02% for nominal 80–95% intervals, and for the Bayesian model, it changed modestly from 58.04–72.94 to 58.82–74.90%. In contrast, the enhanced neural network showed lower WAPE but weaker coverage than the minimal version (53.33–80.78% vs. 61.96–87.06%). Taken together, these results suggest that the richer covariate set generally improved point prediction, while gains in interval performance were model-dependent rather than uniform.

### 4.5 Justification for ensemble averaging with equal weights

Our choice to combine forecasts using a simple unweighted average rather than a single best-performing model or estimated-weight combination was motivated by two distinct considerations from the forecast combination literature. First, this pattern is consistent with longstanding evidence that combining multiple forecasts can reduce model-selection risk and improve robustness across heterogeneous forecasting regimes (17–19). Second, our use of equal weights rather than performance-based weights follows what Stock and Watson (2004) termed the “forecast combination puzzle”: the repeated empirical finding that simple unweighted combinations outperform sophisticated weighted combinations in out-of-sample evaluation (20). Smith and Wallis (2009) attribute this phenomenon to the finite-sample noise introduced when combining weights that are themselves estimated from limited holdout data (21). In our setting, with only 5 holdout years across 51 jurisdictions, estimation error in any data-driven weighting scheme would likely outweigh its theoretical advantage over a simple average. The equal-weighted ensemble, therefore, represents not a default choice, but a principled one grounded in both empirical and theoretical results from the combination forecasting literature.

However, most prior evidence supporting the advantage of simple forecast averaging has come from economics, finance, or general forecasting research rather than public health forecasting. To examine whether this rationale also applied to our setting, we conducted an additional sensitivity analysis using ensemble weights. Specifically, the simple average was compared with four alternative weighting schemes: inverse-RMSE weighting, inverse-MSE weighting, Bates-Granger weighting, and constrained least-squares weighting. The detailed results are presented in Table 6.

**Table 6:** Sensitivity analysis comparing simple and weighted ensemble schemes on the external holdout set.

Weighting scheme	Weights (XGB/NN/Baseline)	Holdout WAPE	Holdout MAE	Holdout MASE	Mean CRPS	Empirical 80% coverage	Empirical 95% coverage	Delta WAPE vs. Simple average
Simple average	0.333/0.333/0.333	20.83%	201.3	5.99	147.86	73.70%	92.20%	+0.00 pp
Bates-Granger	0.072/0.624/0.303	23.14%	223.6	6.21	169.17	70.20%	89.00%	+2.31 pp
Constrained LS	0.089/0.696/0.216	23.06%	222.8	6.13	170.73	69.00%	88.20%	+2.22 pp
Inverse MSE	0.227/0.449/0.324	21.70%	209.7	6.07	159.6	74.50%	90.20%	+0.87 pp
Inverse RMSE	0.278/0.390/0.332	21.25%	205.4	6.03	153.7	72.90%	90.20%	+0.42 pp

### 4.6 Interpretation of state-level forecast patterns

The state-level forecasts suggest that future P&S syphilis burden is likely to remain both concentrated and unevenly distributed across jurisdictions. At the same time, states with the highest projected burden were not necessarily the same as those with the fastest projected growth. This distinction is important because it suggests that future national increases may arise not only from persistently high-burden states, but also from accelerating epidemics in jurisdictions that have not historically contributed the largest share of cases. In practical terms, these patterns imply that absolute burden and growth trajectory capture different dimensions of epidemic risk and may require different public health responses.

### 4.7 Public health implications

The conditional projection of a rise to 131,736 cases by 2033 has implications for public health planning. These projections suggest that continued investment may remain important in historically high-burden states, such as California, Florida, New York, Texas, and Georgia, where the absolute number of projected cases is expected to remain large.

However, the forecasts also point to a second priority: earlier intervention in states with rapid projected growth, even when their current absolute burden is lower. Such jurisdictions may have less established surveillance, prevention, testing, and treatment capacity than long-recognized high-burden areas, and insufficient preparation could further accelerate transmission.

Accordingly, these findings may support a dual planning strategy of sustained control in entrenched high-burden settings and proactive capacity-building in states showing the fastest projected expansion.

### 4.8 Interpretation of predictor importance in the final ensemble

To improve epidemiological interpretation of the final forecasting framework, we conducted a model-agnostic grouped permutation-importance analysis for the final top-3 ensemble on the external holdout set. For each predictor group, we randomly permuted its values across state-year holdout observations while leaving the fitted ensemble unchanged, and then recomputed holdout WAPE; larger increases therefore indicate greater contribution to out-of-sample predictive performance. This analysis showed that recent syphilis history remained the dominant epidemiologic signal in the final ensemble: permuting lag1_log_rate increased WAPE from 20.8 to 26.7% (*Δ* + 5.9 percentage points), whereas permuting lag1_gonorrhea_log_rate and lag1_poverty_rate increased WAPE more modestly, to 22.1% (Δ + 1.3) and 21.8% (Δ + 1.0), respectively, and permuting rolling3_gonorrhea_log_rate increased WAPE to 21.4% (Δ + 0.6). Population had the largest overall effect (20.8 to 90.3%, Δ + 69.5), consistent with its role as a structural scaling input for case-count prediction rather than a standalone epidemiologic driver. Most remaining covariate groups produced near-zero changes in WAPE, suggesting that the final ensemble was driven primarily by recent within-state syphilis momentum, with auxiliary STI and socioeconomic covariates providing secondary refinement (see Table 7).

**Table 7:** Grouped permutation importance of predictors in the final top-3 ensemble on the external holdout set.

Feature group	Permuted WAPE	ΔWAPE (pp)
population_level	90.31%	+69.50
lag1_log_rate	26.71%	+5.90
lag1_gonorrhea_log_rate	22.12%	+1.31
lag1_poverty_rate	21.80%	+0.99
rolling3_gonorrhea_log_rate	21.42%	+0.62
calendar_time	21.36%	+0.55
rolling3_log_rate	21.22%	+0.41
rolling3_hiv_log_rate	20.99%	+0.18
lag1_chlamydia_log_rate	20.92%	+0.11
lag1_poverty_delta	20.91%	+0.10
lag1_income_log_delta	20.88%	+0.07
rolling3_chlamydia_log_rate	20.82%	+0.01
rolling3_hiv_missing_share	20.81%	+0.00
lag1_hiv_missing	20.81%	+0.00
lag1_delta_log_rate	20.80%	−0.01
lag1_hiv_log_rate	20.78%	−0.03
lag1_unemployment_rate	20.65%	−0.16
lag1_unemployment_delta	20.39%	−0.42
lag1_log_median_income	20.23%	−0.58

### 4.9 Strengths

This study has several strengths. First, it compared multiple candidate forecasting approaches rather than relying on a single model class, thereby reducing the risk that the conclusions were driven by a single modeling framework. Second, the use of an ensemble reduced model-selection uncertainty while preserving near-best predictive performance. Third, model evaluation combined external holdout testing, rolling-origin backtesting, probabilistic metrics, and spatial diagnostics, providing a broader assessment of predictive performance than any single accuracy measure. Finally, the study addresses an important gap in the US syphilis forecasting literature by providing explicit forecasts at both the state and national levels across all 50 states and the District of Columbia.

### 4.10 Limitations

Several limitations should be considered when interpreting these forecasts. First, although the models incorporated observed surveillance history and multiple covariates, the covariate values over the forecast horizon were extrapolated rather than observed, introducing additional uncertainty into the long-range projections. Second, probabilistic calibration was imperfect. In particular, interval calibration relied on residuals from the relatively short 2017–2018 calibration window, so extrapolation to future years may be sensitive to distribution shift if the relationship between calibration-period residuals and future forecast uncertainty changes over time. Third, the modeling framework did not explicitly encode structural breaks such as policy interventions, changes in screening or reporting, or shifts in sexual behavior and prevention uptake. Accordingly, the forecasts should be interpreted as projections conditional on broad continuation of the main patterns represented in the historical data rather than as predictions of abrupt regime change. In addition, forecast uncertainty is expected to increase with the forecast horizon because recursive forecasting may accumulate prediction errors over time, and long-range projections may be sensitive to future changes in transmission dynamics, surveillance and reporting practices, policy interventions, prevention uptake, and extrapolated covariate trajectories.

Several additional limitations of the evaluation design should also be noted. First, the fixed external holdout covered only 2019–2023. Although this corresponded to 51 jurisdictions over five annual test years and was complemented by rolling-origin backtesting across four forecast origins and three forecast horizons, it still represented a limited fixed-holdout window relative to the 10-year projection horizon. Extending the fixed holdout further would have necessarily reduced the data available for model fitting and interval calibration. Second, the Bayesian negative binomial model was not included in rolling-origin backtesting because repeated posterior sampling across multiple expanding windows was computationally intensive; therefore, robustness comparisons across model families were not fully balanced. However, the impact of this imbalance on the primary practical conclusion is likely more limited because the Bayesian specification was not retained in the final forecasting framework. Its inclusion was intended to broaden the methodological comparison and to provide an empirical reference for future studies considering hierarchical Bayesian forecasting for similarly structured state-level data and long recursive forecast horizons. Third, the pairwise Diebold–Mariano tests were applied to pooled state-year forecast errors and should therefore be interpreted cautiously given the panel structure of the evaluation data.

Additional limitations apply to interpretation at the national level and to the spatial structure of the final forecasts. National coverage estimates were evaluated on only five holdout years (*n* = 5), which limits the statistical reliability of that assessment. In addition, although explicit spatial modeling was explored through a BYM2/CAR Bayesian specification, the final ensemble did not include an explicit BYM2/CAR spatial model; therefore, the final ensemble forecasts do not directly inherit that class of explicit spatial smoothing structure. These considerations do not negate the utility of the forecasts, but they do suggest caution when using them for fine-grained probabilistic interpretation, especially for interval calibration, or in scenarios where future transmission dynamics may depart materially from the historical record.

## 5 Conclusion

In conclusion, this study provides a practical and competitive framework for state-level forecasting of primary and secondary syphilis in the United States. Across the external holdout period, the final top-3 global ensemble achieved competitive overall performance, while XGBoost emerged as the best-performing individual model and the top-ranked ensemble member. More broadly, the selected enhanced machine-learning models outperformed the autoregressive baselines, suggesting that incorporating richer epidemiologic and contextual information can improve predictive accuracy beyond simple historical carry-forward dynamics alone. When refit for future projection, the final ensemble suggested a continued rise in national burden, reaching approximately 131,736 cases in 2033. Taken together, these findings support the value of data-driven state-level forecasting for anticipating substantial future burden and for supporting preparedness and sustained response planning, while also underscoring that such projections should be interpreted alongside their uncertainty and modeling assumptions.

## Data Availability

All data supporting the findings of this study have been deposited in the public Zenodo repository and are available at: https://doi.org/10.5281/zenodo.21352148.
