# Supplementary material for: Forecasting continued increase in primary and secondary syphilis in the United States, 2024–2033: a state-level multi-model ensemble study
Source: Front Public Health. 2026 Jul 24;14:1888674. doi: 10.3389/fpubh.2026.1888674 (PMC13447349; doi:10.3389/fpubh.2026.1888674)
Supplement: Supplementary file 1 [file Data_Sheet_1.DOCX]

**Supplementary Text S1. Additional implementation details for preprocessing, feature construction, interval calibration, and national aggregation**

# 1. Notation, modeling units, and time splits

Let $i$index jurisdiction and $t$index calendar year. For jurisdiction $i$in year $t$, let $C_{it}$denote the annual number of reported primary and secondary (P&S) syphilis cases, and let $P_{it}$denote the corresponding population size. The observed annual rate per 100,000 population was defined as:

$$r_{it}=100,000\times\frac{C_{it}}{P_{it}},$$

and the log-transformed syphilis rate used by the non-Bayesian forecasting models was defined as:

$$y_{it}=\log\left( 1+r_{it} \right).$$

The state-level modeling units were the 50 U.S. states plus the District of Columbia ($n=51$). A national reference row with area_id = 00 was retained in the processed panel, but it was not treated as an additional independent modeling unit in state-level model fitting, hyperparameter tuning, model-comparison metrics, rolling-origin backtesting, or residual diagnostics. National forecasts were instead generated only after state-level prediction by summing jurisdiction-level predicted counts within year.

The observed data covered 2000-2023. Years 2000-2002 were retained only for lag construction. In the standard main run, 2003-2016 served as the internal training period for the non-Bayesian models, 2017-2018 served as the internal calibration period for residual-based interval construction and selected tuning steps, 2019-2023 was the external holdout period, and 2024-2033 was the future forecast period. Rolling-origin backtesting for the non-Bayesian models used forecast origins at 2012, 2014, 2016, and 2018.

# 2. Shared preprocessing

Raw case and population fields were first coerced to numeric values, after which annual rates and log-rates were recomputed from the cleaned quantities using the definitions above. State/DC records were then aligned by jurisdiction-year with auxiliary series for gonorrhea, chlamydia, HIV diagnoses, poverty, unemployment, and median household income. For the syphilis outcome panel and STI covariate panels, the national reference row was generated by summing state counts and populations by year and recomputing the corresponding national rate; for socioeconomic covariates, national annual values were constructed as population-weighted averages across states.

For XGBoost, the neural network, and the Bayesian fixed-effect design matrix, numeric preprocessing followed a common workflow. Numeric predictors were median-imputed using statistics estimated from the relevant fitting subset. Whenever a numeric predictor contained missing values in the fitting data, the preprocessing pipeline automatically created a binary missingness indicator of the form feature_was_missing. The completed numeric design matrix was then standardized by z-score scaling.

For XGBoost and the neural network, area_id was additionally encoded as a one-hot categorical input, with one level dropped and unknown categories ignored at prediction time. For the Bayesian negative binomial model, area_id was not one-hot encoded; jurisdiction-level heterogeneity was instead represented through hierarchical random effects and spatial structure.

# 3. Shared feature construction

For each jurisdiction-year, the pipeline constructed lagged syphilis-history predictors from the previous three annual log-rates:

$$y_{i,t-1},y_{i,t-2},y_{i,t-3}.$$

From these values, the core temporal features were

$$\text{lag1\_log\_rate}_{it}=y_{i,t-1},$$

$$\text{rolling3\_log\_rate}_{it}=\frac{1}{3}\left( y_{i,t-1} + y_{i,t-2} + y_{i,t-3} \right),$$

and

$$\text{lag1\_delta\_log\_rate}_{it}=y_{i,t-1}-y_{i,t-2}.$$

A calendar-time index was also included:

$$\text{year\_idx}_{it}=t-2000.$$

For the non-Bayesian models, the population term was represented as

$$\text{log\_population}_{it}=\log\left( P_{it} \right).$$

The minimal feature set was

$$x_{it}^{(min)}=\left( \text{year\_idx}_{it},\text{lag1\_log\_rate}_{it},\text{rolling3\_log\_rate}_{it},\text{lag1\_delta\_log\_rate}_{it},\text{log\_population}_{it},\text{is\_national}_{it} \right).$$

The enhanced feature set extended the minimal feature set by adding lagged and rolling gonorrhea and chlamydia log-rates, lagged socioeconomic indicators and their annual changes, and HIV-related predictors including missingness indicators:

$$x_{it}^{\left( \text{enh} \right)}=(x_{it}^{(min)},\text{lag1\_gonorrhea\_log\_rate}_{it},\text{rolling3\_gonorrhea\_log\_rate}_{it},\text{lag1\_chlamydia\_log\_rate}_{it},\text{rolling3\_chlamydia\_log\_rate}_{it},$$

$$\text{lag1\_poverty\_rate}_{it},\text{lag1\_unemployment\_rate}_{it},\text{lag1\_log\_median\_income}_{it},\text{lag1\_poverty\_delta}_{it},\text{lag1\_unemployment\_delta}_{it},\text{lag1\_income\_log\_delta}_{it},$$

$$\text{lag1\_hiv\_log\_rate}_{it},\text{rolling3\_hiv\_log\_rate}_{it},\text{lag1\_hiv\_missing}_{it},\text{rolling3\_hiv\_missing\_share}_{it}).$$

For the Bayesian model, the corresponding minimal and enhanced fixed-effect feature vectors were identical except that log_population was removed because population entered the model through an offset.

# 4. Future population and covariate extrapolation

Population was extended through 2033 before recursive future forecasting. In the standard main run, state population values from 2014-2023 were extrapolated on the log scale using a log-linear trend and then exponentiated back to the original scale. The projected state populations were summed to obtain the national reference population for each future year.

The standard main run used linear covariate extrapolation. The scale of extrapolation depended on variable type. Gonorrhea, chlamydia, and HIV-related rate variables were extrapolated on the $\log(1+\text{rate})$scale and then back-transformed. Median household income was extrapolated on the log scale and then exponentiated. Poverty and unemployment were extrapolated on the original scale and clipped to $\left[ 0 , 100 \right]$. These projected future covariates were then used to construct recursive features for 2024-2033.

(Note: In the current implementation, these extrapolated future covariate values were treated as fixed deterministic inputs during recursive forecasting rather than as stochastic quantities with their own predictive distributions. Accordingly, uncertainty in future covariate extrapolation was not explicitly propagated into the reported prediction intervals. The reported intervals instead reflect residual-based calibration uncertainty for the non-Bayesian models, posterior predictive uncertainty for the Bayesian model, and mixture-sampling uncertainty for the final ensemble.)

# 5. Candidate models

**State-specific linear trend baseline**[1]

The state-specific linear trend baseline was specified separately for each state as a regression-based linear time-trend model on the log-rate scale.

The state-specific linear trend baseline was specified separately for each state as a linear time-trend model on the log-rate scale:

$$y_{it}=\alpha_{i}+\beta_{i}t+\varepsilon_{it},$$

where $y_{it}$is the log-transformed syphilis rate for state $i$in year $t$, $t$denotes calendar time, $\alpha_{i}$is the state-specific intercept, $\beta_{i}$is the state-specific linear time-trend coefficient, and $\varepsilon_{it}$is the residual term. This model extrapolates future burden solely from the historical linear trend in syphilis log-rates and does not incorporate additional covariates. In the current pipeline, the state-specific linear trend baseline was treated as a single baseline model rather than as substantively distinct minimal and enhanced variants.

In the standard main run, jurisdiction-specific linear trends were estimated whenever sufficient within-jurisdiction data were available. If a jurisdiction-specific fit could not be estimated, the slope was set to zero and the intercept was set to the available jurisdiction-specific mean log-rate. A pooled global linear trend was retained as a final safeguard.

## 5.1. AR(1)

The AR(1) baseline model was specified in mean-corrected form as[2]

$$y_{it}-\mu_{i}=\phi_{i}\left( y_{i,t-1} - \mu_{i} \right)+\varepsilon_{it},\mid\phi_{i}\mid<1,$$

where $i$indexes state and $t$indexes calendar year; $y_{it}$is the log-transformed syphilis rate for state $i$in year $t$; $y_{i,t-1}$is the corresponding log-rate in the previous year; $\mu_{i}$is the mean training-period log-rate for state $i$; $\phi_{i}$is the first-order autoregressive coefficient; and $\varepsilon_{it}$is the residual term. Under this parameterization, the current year’s deviation from the state-specific mean, $y_{it}-\mu_{i}$, is modeled as proportional to the previous year’s deviation, $y_{i,t-1}-\mu_{i}$. The stationarity constraint $\mid\phi_{i}\mid<1$was imposed to prevent long-horizon recursive divergence. As with the state-specific linear trend baseline, the AR(1) model was handled as a single baseline specification and did not differ substantively between the minimal and enhanced branches of the pipeline.

In the standard main run, jurisdiction-specific coefficients were estimated when sufficient lagged observations were available. The fitted autoregressive coefficient was clipped to $\left[ -0.95,0.95 \right]$before recursive forecasting. When sufficient jurisdiction-specific lagged information was not available, the implementation fell back to a jurisdiction-specific mean model, which is equivalent to setting $\varphi_{i}$= 0; pooled global coefficients were retained as a safeguard if a jurisdiction-specific coefficient set was unavailable at prediction time.

## 5.2. XGBoost

**Minimal model[3]**

The minimal XGBoost model predicted the syphilis log-rate as

$$\hat{y}_{it}=f_{\mathrm{XGB}}(x_{it}^{(min)},z_{i})=\sum_{m=1}^{M} f_{m}(x_{it}^{(min)},z_{i}),$$

where $x_{it}^{(min)}$is the minimal numeric feature vector for state $i$in year $t$, $z_{i}$is the one-hot encoded state identifier, $f_{m}(\cdot)$is the $m$-th regression tree, and $M$is the total number of trees in the boosted ensemble. In the minimal specification, $x_{it}^{(min)}$included the year index, the previous-year syphilis log-rate, the recent 3-year rolling mean log-rate, the previous-year change in syphilis log-rate, and log-population. The inclusion of $z_{i}$allowed the model to capture state-specific baseline differences.

**Enhanced model**

The enhanced XGBoost model retained the same functional form,

$$\hat{y}_{it}=f_{\mathrm{XGB}}(x_{it}^{\left( \text{enh} \right)},z_{i})=\sum_{m=1}^{M} f_{m}(x_{it}^{\left( \text{enh} \right)},z_{i}),$$

but replaced the minimal numeric feature vector with an enhanced vector $x_{it}^{\left( \text{enh} \right)}$. In addition to the minimal features, $x_{it}^{\left( \text{enh} \right)}$included lagged and rolling gonorrhea log-rates, lagged and rolling chlamydia log-rates, lagged poverty rate, lagged unemployment rate, lagged log median household income, the corresponding annual changes in poverty, unemployment, and income, and lagged and rolling HIV log-rates together with HIV missingness indicators. The one-hot encoded state identifier $z_{i}$was retained in the enhanced model.

In the standard main run, XGBoost used squared-error regression on the log-rate scale (objective = "reg:squarederror"), with random_state = 20260510 and n_jobs = 4. Two candidate hyperparameter sets were evaluated:

$$(n_{\text{estimators}},\text{max\_depth},\text{learning\_rate},\text{subsample},\text{colsample\_bytree},\text{reg\_lambda})=(220,2,0.05,0.90,0.90,1.0),$$

and

$$(n_{\text{estimators}},\text{max\_depth},\text{learning\_rate},\text{subsample},\text{colsample\_bytree},\text{reg\_lambda})=(320,3,0.03,0.85,0.85,2.0).$$

The retained parameter set was selected using recursive predictions over the 2017-2018 calibration period, with calibration-period WAPE as the selection criterion.

## 5.3. Neural Network

**Minimal model[4, 5]**

The minimal neural network model was a two-hidden-layer feed-forward network:

$$h_{it}^{\left( 1 \right)}=g\text{ }\left( W_{1}[x_{it}^{(min)},z_{i}]+b_{1} \right),$$

$$h_{it}^{\left( 2 \right)}=g\text{ }\left( W_{2}h_{it}^{\left( 1 \right)} + b_{2} \right),$$

$$\hat{y}_{it}=W_{3}h_{it}^{\left( 2 \right)}+b_{3},$$

where $\hat{y}_{it}$is the predicted log-transformed syphilis rate for state $i$in year $t$; $x_{it}^{(min)}$is the minimal numeric feature vector; $z_{i}$is the one-hot encoded jurisdiction identifier; $\left[ x_{it}^{(min)} , z_{i} \right]$denotes concatenation of the numeric and categorical inputs; $h_{it}^{\left( 1 \right)}$and $h_{it}^{\left( 2 \right)}$are the first and second hidden-layer representations; $W_{1}$, $W_{2}$, and $W_{3}$are weight matrices; $b_{1}$, $b_{2}$, and $b_{3}$are bias terms; and $g(\cdot)$denotes the ReLU activation function. In the implemented architecture, the two hidden layers contained 96 and 48 neurons, respectively. The minimal numeric feature vector included the year index, the previous-year syphilis log-rate, the recent 3-year rolling mean log-rate, the previous-year change in log-rate, and log-population. Jurisdiction identifiers were additionally included as one-hot encoded categorical features to allow the model to capture state-specific baseline differences. Dropout regularization was applied after each hidden layer.

**Enhanced model**

The enhanced neural network retained the same architecture:

$$h_{it}^{\left( 1 \right)}=g\text{ }\text{ }\left( W_{1}[x_{it}^{\left( \text{enh} \right)},z_{i}]+b_{1} \right),$$

$$h_{it}^{\left( 2 \right)}=g\text{ }\text{ }\left( W_{2}h_{it}^{\left( 1 \right)} + b_{2} \right),$$

$$\hat{y}_{it}=W_{3}h_{it}^{\left( 2 \right)}+b_{3},$$

where $\hat{y}_{it}$, $z_{i}$, $h_{it}^{\left( 1 \right)}$, $h_{it}^{\left( 2 \right)}$, $W_{1}$, $W_{2}$, $W_{3}$, $b_{1}$, $b_{2}$, $b_{3}$, and $g(\cdot)$have the same interpretation as in the minimal model, while $x_{it}^{\left( \text{enh} \right)}$denotes the enhanced numeric feature vector. Compared with the minimal specification, $x_{it}^{\left( \text{enh} \right)}$additionally incorporated STI, socioeconomic, and HIV-related predictors. The one-hot encoded state identifier $z_{i}$and dropout regularization were retained in the enhanced model.

In the standard main run, the implemented architecture used hidden-layer widths of 96 and 48, dropout rates of 0.10 and 0.05, AdamW optimization, learning rate $0.0015$, weight decay ${10}^{-4}$, a maximum of 320 epochs, and early-stopping patience of 40 epochs. The target $y_{it}$was standardized within the training subset and transformed back after prediction.

## 5.4. Bayesian Negative Binomial (BYM2/CAR)

**Minimal model**

The minimal Bayesian count model was specified as

$$C_{it}\mid\mu_{it},\alpha\sim\mathrm{NegBin}(\mu_{it},\alpha),$$

$$\log(\mu_{it})=\log(P_{it}/100,000)+\beta_{0}+\left( x_{it}^{\left( \min,B \right)} \right)^{T}\beta+u_{i},$$

where $C_{it}$is the observed annual case count, $\mu_{it}$is the expected annual case count, $\alpha$is the overdispersion parameter[6], $\alpha$is the overdispersion parameter, $\beta_{0}$is the intercept, $\beta$is the regression coefficient vector, $x_{it}^{\left( \min,B \right)}$is the minimal Bayesian feature vector, and $u_{i}$is the state-level random effect. The term $\log(P_{it}/100,000)$is a population offset[7], so log-population was not entered as an ordinary predictor in the Bayesian model.

The state-level random effect followed a BYM2/CAR-type decomposition[8]:

$$u_{i}=\sigma_{u}\left[ \sqrt{1-\rho}\text{ }v_{i} + \sqrt{\rho}\text{ }s_{i} \right],$$

where $\sigma_{u}$is the overall scale of the state effect, $\rho$controls the proportion of spatially structured variation, $v_{i}$is the unstructured state-specific random effect, and $s_{i}$is the structured spatial effect defined through the state adjacency matrix. In the Bayesian formulation, prior distributions were assigned to the unknown parameters, and inference was based on the posterior distribution

$$p(\theta\mid C)\propto\left[ \prod_{i,t} \mathrm{NegBin}(C_{it}\mid\mu_{it},\alpha) \right]p(\theta),$$

where $\theta$denotes the collection of unknown model parameters and random effects, and $p(\theta)$denotes their joint prior distribution. Forecasts and uncertainty intervals were then obtained from the posterior predictive distribution. The minimal Bayesian feature vector $x_{it}^{\left( \min,B \right)}$included the year index, the previous-year syphilis log-rate, the recent 3-year rolling mean log-rate, and the previous-year change in log-rate.

**Enhanced model**

The enhanced Bayesian model retained the same negative binomial likelihood and Bayesian hierarchical structure:

$$C_{it}\mid\mu_{it},\alpha\sim\mathrm{NegBin}(\mu_{it},\alpha),$$

$$\log(\mu_{it})=\log(P_{it}/100,000)+\beta_{0}+\left( x_{it}^{\left( \text{enh} , B \right)} \right)^{T}\beta+u_{i},$$

with the same interpretation of $C_{it}$, $\mu_{it}$, $\alpha$, $\beta_{0}$, $\beta$, $u_{i}$, and the population offset. Here, $x_{it}^{\left( \text{enh} , B \right)}$extended the minimal Bayesian feature vector by adding lagged and rolling gonorrhea and chlamydia log-rates, lagged socioeconomic indicators and their annual changes, and HIV-related predictors including missingness indicators. The same BYM2/CAR spatial random-effect structure was used:

$$u_{i}=\sigma_{u}\left[ \sqrt{1-\rho}\text{ }v_{i} + \sqrt{\rho}\text{ }s_{i} \right].$$

As in the minimal Bayesian model, prior distributions were assigned to the unknown parameters, posterior inference combined the negative binomial likelihood with these priors, and forecasts with uncertainty intervals were derived from the posterior predictive distribution.

In the standard main run, posterior sampling used PyMC NUTS with 500 posterior draws per chain, 500 tuning iterations per chain, 4 chains, 1 core, and target_accept = 0.95. The fixed-effect design matrix used the shared numeric preprocessing described above, but did not include one-hot encoded area_id.

Additional prior settings for the Bayesian negative binomial model used in the standard main run are summarized in Table 1.

Table 1 Prior specifications for the enhanced Bayesian negative binomial model

| **Component** | **Symbol / parameter** | **Specification in the standard main run** | **Prior type / support** |
| --- | --- | --- | --- |
| Default fixed-effect prior | $\beta_{j}^{\left( \mathrm{raw} \right)}$ | $\mathrm{Normal}(0,1.5)$ | Weakly informative prior |
| Prior for lagged syphilis term | $\beta_{\mathrm{lag}1}$ | $\mathrm{Normal}(0.7s_{\mathrm{lag}1},0.15s_{\mathrm{lag}1})$ | Conservative Minnesota-type lag prior [9] |
| Prior for rolling 3-year mean term | $\beta_{\mathrm{rolling}3}$ | Normal(0,1.0_srolling3_) | weakly informative prior |
| Overdispersion prior | $\alpha$ | $\mathrm{Exponential}(1.0)$ | Weakly informative prior |
| Area-effect scale prior | $\sigma_{u}$ | $\mathrm{HalfNormal}(1.0)$ | Weakly informative prior |
| Spatial mixing prior | $\rho$ | $\mathrm{Beta}(0.5,0.5)$ | Bounded BYM2 mixing hyperprior; consistent with recent small-area BYM2 applications assigning $\mathrm{Beta}(0.5,0.5)$to the spatial variance proportion parameter, which controls the structured versus unstructured random-effect variance contribution[10]. |
| Unstructured area effect | $v_{i}/u_{\mathrm{raw}}$ | $\mathrm{Normal}(0,1)$ | Weakly informative prior |
| Spatial dependence prior | spatial_alpha | Beta(1.0,1.0) | Bounded weakly informative；The prior assigned to spatial_alpha was specified as $\mathrm{Beta}(1.0,1.0)$, which is equivalent to a uniform distribution on the unit interval $\left[ 0 , 1 \right]$. This is consistent with common CAR model specifications in which the spatial dependence parameter is constrained to lie between 0 and 1. For example, the CARBayes documentation describes the Leroux CAR spatial dependence parameter $\rho$as taking values in the unit interval and assigns it a $\mathrm{Uniform}(0,1)$prior. Therefore, the $\mathrm{Beta}(1.0,1.0)$prior used here can be interpreted as a bounded uniform, weakly informative prior for the CAR spatial dependence parameter[11]. |

Because strong prior information was limited for most model components, the Bayesian specification used predominantly weakly informative or regularizing priors, with more targeted informative priors applied only to selected lag terms. Sensitivity analyses indicated some dependence on the lag1, rolling3, and ρ prior settings, particularly in the minimal specification, whereas sensitivity to the spatial_alpha prior was comparatively small. However, the magnitude of these changes was within an acceptable range for the present robustness analysis, because they did not alter the qualitative interpretation of the Bayesian model, which remained weaker than the leading non-Bayesian candidates. Detailed prior-sensitivity results are provided in Table 2.

Table 2 Prior-sensitivity analysis for the Bayesian negative binomial model

| **Prior family** | **Feature set** | **Main/original setting** | **Main/original WAPE** | **Sensitivity setting 1** | **Sensitivity WAPE 1** | **Sensitivity setting 2** | **Sensitivity WAPE 2** |
| --- | --- | --- | --- | --- | --- | --- | --- |
| Lag-1 coefficient prior | Enhanced | Normal(mean = 0.70, sd = 0.15) | 65.60% | Normal(mean = 0.50, sd = 0.20) | 66.76% | Normal(mean = 0.90, sd = 0.10) | 67.12% |
| Lag-1 coefficient prior | Minimal | Normal(mean = 0.70, sd = 0.15) | 74.31% | Normal(mean = 0.50, sd = 0.20) | 72.38% | Normal(mean = 0.90, sd = 0.10) | 72.75% |
| Rolling-3 coefficient prior | Enhanced | Normal(0; 1.0 × feature SD) | 65.60% | Normal(0; 0.5 × feature SD) | 66.67% | Normal(0; 1.5 × feature SD) | 67.20% |
| Rolling-3 coefficient prior | Minimal | Normal(0; 1.0 × feature SD) | 74.31% | Normal(0; 0.5 × feature SD) | 71.69% | Normal(0; 1.5 × feature SD) | 73.85% |
| BYM2 mixing prior ((\rho)) | Enhanced | Beta(0.5, 0.5) | 65.60% | Beta(1.0, 1.0) | 66.83% | Beta(2.0, 2.0) | 67.27% |
| BYM2 mixing prior ((\rho)) | Minimal | Beta(0.5, 0.5) | 74.31% | Beta(1.0, 1.0) | 73.45% | Beta(2.0, 2.0) | 73.58% |
| CAR spatial dependence prior (spatial_alpha) | Enhanced | Beta(1.0, 1.0) | 65.60% | Beta(2.0, 1.0) | 66.68% | — | — |
| CAR spatial dependence prior (spatial_alpha) | Minimal | Beta(1.0, 1.0) | 74.31% | Beta(2.0, 1.0) | 74.48% | — | — |

# 6. Hyperparameter tuning and internal model selection

All hyperparameter tuning and internal model-development steps were confined to the pre-holdout period. In the state-level modeling panel, 2003–2016 (14 annual modeling years; 714 state-year observations across 51 jurisdictions) served as the proper internal fitting subset, and 2017–2018 (2 annual years; 102 state-year observations) served as the internal calibration/validation subset. The final pre-holdout fitting period used for the retained models was 2003–2018 (16 annual years; 816 state-year observations). No information from the 2019–2023 external holdout period was used for hyperparameter selection, early stopping, or interval calibration.

For XGBoost, hyperparameter tuning was performed by comparing two prespecified candidate parameter sets in the standard main run. The first candidate was (n_estimators = 220, max_depth = 2, learning_rate = 0.05, subsample = 0.90, colsample_bytree = 0.90, reg_lambda = 1.0), and the second was (n_estimators = 320, max_depth = 3, learning_rate = 0.03, subsample = 0.85, colsample_bytree = 0.85, reg_lambda = 2.0). Both candidates used squared-error regression (objective = "reg:squarederror"), random_state = 20260510, and n_jobs = 4. Each candidate model was first fit on the 2003–2016 internal fitting subset, then evaluated by recursive prediction over 2017–2018, and calibration-period weighted absolute percentage error (WAPE) on predicted case counts was used as the selection criterion. After the lower-WAPE candidate was selected, that parameter setting was retained and the final XGBoost model was re-fit on the full 2003–2018 fitting data. Residual-based interval calibration for XGBoost was based on the same 2017–2018 recursive predictions, using the empirical 10th/90th and 2.5th/97.5th quantiles of the log-rate residuals.

For the neural network, we did not perform a multi-candidate grid search. Instead, the architecture and optimization settings were fixed in advance and then internally validated using the 2017–2018 subset for early stopping. In the standard main run, the model was a two-hidden-layer feed-forward network with hidden-layer widths of 96 and 48 units, ReLU activation, dropout rates of 0.10 and 0.05, AdamW optimization, learning rate = 0.0015, weight_decay = 1e-4, maximum epochs = 320, and early-stopping patience = 40 epochs. The global random seed was fixed at 20260510. The target log-rate was standardized using the mean and standard deviation estimated from the 2003–2016 internal fitting subset, and the 2017–2018 subset was supplied as the validation set during training. After internal validation, the final neural network was re-trained on the full 2003–2018 fitting data using the same fixed architecture and optimization settings. As with XGBoost, residual-based interval calibration for the neural network used the 2017–2018 recursive predictions and the empirical 10th/90th and 2.5th/97.5th residual quantiles.

The naive drift and AR(1) baselines did not involve hyperparameter tuning. Both were first estimated on the 2003–2016 internal fitting subset, then internally calibrated on the 2017–2018 recursive predictions, and finally re-fit on the full 2003–2018 fitting period before external holdout evaluation and future forecasting. For AR(1), the fitted autoregressive coefficient was clipped to the interval [-0.95, 0.95] to enforce long-horizon recursive stability; this was a fixed implementation constraint rather than a tuned hyperparameter.

The Bayesian negative binomial model likewise did not undergo predictive hyperparameter search against the 2017–2018 subset. Instead, its sampler configuration and prior settings were fixed in advance in the standard main run. Posterior sampling used PyMC NUTS with 500 posterior draws per chain, 500 tuning iterations per chain, 4 chains, 1 core, and target_accept = 0.95. Because Bayesian uncertainty intervals were obtained from the posterior predictive distribution rather than from residual-based conformal calibration, the Bayesian model was fit directly on the full 2003–2018 fitting data rather than entering the non-Bayesian calibration workflow. Robustness of the Bayesian specification was assessed separately through prior sensitivity analyses rather than through calibration-period hyperparameter search.

**Reference**

1.	Hyndman R J, Athanasopoulos G. Forecasting: Principles and Practice. In: Book Forecasting: Principles and Practice [Internet]. Melbourne, Australia: OTexts; 2021 [updated Date; cited Access Date]. Available from: <https://otexts.com/fpp3/>.

2.	Hyndman R J, Athanasopoulos G. Forecasting: Principles and Practice. In: Book Forecasting: Principles and Practice [Internet]. Melbourne, Australia: OTexts; 2021 [updated Date; cited Access Date]. Available from: <https://otexts.com/fpp3/AR.html>.

3.	Chen T, Guestrin C, editors. Xgboost: A scalable tree boosting system. Proceedings of the 22nd acm sigkdd international conference on knowledge discovery and data mining; 2016.

4.	Zhang A, Lipton Z C, Li M, Smola A J. Dive into Deep Learning [Internet]. Dive into Deep Learning; 2023 [cited 21 May 2026]. Available from: <https://d2l.ai/chapter_multilayer-perceptrons/mlp.html>

5.	Goodfellow I, Bengio Y, Courville A. Deep Feedforward Networks. In: Deep Learning [Internet]. Cambridge, MA: MIT Press; 2016 [updated cited 21 May 2026]. Available from: <https://www.deeplearningbook.org/contents/mlp.html>.

6.	Hilbe J M. Negative Binomial Regression. Cambridge: Cambridge University Press; 2007.

7.	Parry S. To Offset or Not: Using Offsets in Count Models. 2022 April 2022.

8.	Morris M. Spatial Models in Stan: Intrinsic Auto-Regressive Models for Areal Data [Internet]. Stan; 2019 [cited 21 May 2026]. Available from: <https://mc-stan.org/learn-stan/case-studies/icar_stan.html>

9.	LLC S. bayes: var — Bayesian vector autoregressive models [Internet]. College Station, TX: Stata Press / StataCorp LLC; 2025 [cited 19 May 2026]. Available from: <https://www.stata.com/manuals/bayesbayesvar.pdf>

10.	Okonek T, Wakefield J. A Computationally Efficient Approach to Fully Bayesian Benchmarking. Journal of Official Statistics. 2024;40(2):283–316. doi:10.1177/0282423x241244670

11.	Lee D. CARBayes version 6.1.1: An R Package for Spatial Areal Unit Modelling with Conditional Autoregressive Priors. CRAN; 2024.
