# Supplementary material for: Forecasting continued increase in primary and secondary syphilis in the United States, 2024–2033: a state-level multi-model ensemble study
Source: Front Public Health. 2026 Jul 24;14:1888674. doi: 10.3389/fpubh.2026.1888674 (PMC13447349; doi:10.3389/fpubh.2026.1888674)
Supplement: Supplementary file 2 [file Data_Sheet_2.DOCX]

**Supplementary Text S2. Sensitivity analysis of residual-calibration window for the final top-3 ensemble**

**1. Purpose of the sensitivity analysis**

In the main analysis, prediction intervals for the non-Bayesian models were constructed using a residual-based calibration approach. The original residual-calibration window was 2017–2018. Because this window was relatively short, we conducted an additional sensitivity analysis to examine whether using alternative, longer calibration windows would materially change the performance of the final top-3 ensemble.

The purpose of this analysis was not to reselect the final forecasting model, but to evaluate whether the main results were sensitive to the choice of calibration window used for residual-based interval construction.

**2. Alternative calibration windows**

We compared the original calibration window, 2017–2018, with two alternative calibration windows:

1. 2016–2018 calibration window
2. 2015–2018 calibration window

For each alternative calibration window, the residual-calibration procedure was repeated and the final top-3 ensemble was re-evaluated on the same external holdout period. Other major procedures and parameter settings were kept consistent with the main analysis. Because extending the calibration window requires assigning more pre-holdout years to calibration, it also reduces the number of years available for model training. Therefore, the sensitivity analysis reflects the trade-off between using more years for residual calibration and preserving sufficient historical data for model fitting.

**3. Results**

The calibration-window sensitivity analysis showed that extending the calibration window affected some evaluation metrics, but the changes were not uniformly favorable.

Under the 2015–2018 calibration window, several indicators showed modest improvement compared with the original 2017–2018 calibration window. The final top-3 ensemble WAPE decreased from 20.83% to 19.10%, corresponding to an absolute decrease of 1.73 percentage points and a relative decrease of 8.30%. MAE decreased from 201.33 to 184.63, and MASE decreased slightly from 5.99 to 5.90. Empirical coverage also improved. The 80% prediction-interval coverage increased from 76.08% to 80.39%, reaching approximately the nominal 80% level. The 95% prediction-interval coverage increased from 90.98% to 93.33%, although it remained slightly below the nominal 95% level.

However, the improvement under the 2015–2018 calibration window was not uniform across all metrics. Mean CRPS increased slightly from 149.03 to 149.24. This indicates that although several point-forecast and empirical-coverage indicators improved, the overall probabilistic scoring metric did not improve under this longer calibration window.

Under the 2016–2018 calibration window, the results were more mixed. WAPE decreased slightly from 20.83% to 20.26%, and MAE decreased from 201.33 to 195.82. However, several other indicators worsened. MASE increased from 5.99 to 6.13, mean CRPS increased from 149.03 to 153.51, and 80% empirical coverage decreased from 76.08% to 74.51%. Although 95% empirical coverage increased slightly from 90.98% to 91.76%, it remained below the nominal 95% level.

**Table S2. Sensitivity analysis of calibration-window choice for the final top-3 ensemble**

| **Calibration window** | **WAPE** | **MAE** | **MASE** | **Mean CRPS** | **80% coverage** | **95% coverage** |
| --- | --- | --- | --- | --- | --- | --- |
| 2017–2018, original | 20.83% | 201.33 | 5.99 | 149.03 | 76.08% | 90.98% |
| 2016–2018 | 20.26% | 195.82 | 6.13 | 153.51 | 74.51% | 91.76% |
| 2015–2018 | 19.10% | 184.63 | 5.90 | 149.24 | 80.39% | 93.33% |

**4. Interpretation**

Overall, the sensitivity analysis suggests that the residual-calibration window can influence both point-forecast and interval-calibration metrics. The 2015–2018 calibration window produced modest improvements in WAPE, MAE, MASE, and empirical coverage. However, it did not improve mean CRPS. The 2016–2018 calibration window improved WAPE and MAE slightly, but worsened MASE, mean CRPS, and 80% empirical coverage. Therefore, extending the calibration window did not automatically or uniformly improve overall predictive performance.

These results suggest that a longer calibration window may help empirical coverage in some cases, but it may also introduce trade-offs. A longer window provides more residuals for empirical interval calibration, but it also reduces the amount of data available for model training. In addition, residual-based calibration may have a more direct effect on near-term holdout performance than on long-horizon forecasts. As the forecast horizon extends to 2033, the corrective effect of a fixed historical calibration window may become less direct because future errors may increasingly reflect distributional shifts, recursive error accumulation, and uncertainty in extrapolated covariates.

**5. Conclusion**

The sensitivity analysis indicates that the original 2017–2018 residual-calibration strategy was not invalidated by the alternative calibration windows. The 2015–2018 window improved several indicators, especially empirical coverage, but the improvement was not uniform across all metrics. The 2016–2018 window showed mixed results, with some indicators improving and others worsening. Therefore, we retained the original calibration strategy in the main analysis while acknowledging calibration-window choice as an important source of uncertainty.

The prediction intervals should therefore be interpreted as approximate empirical prediction intervals rather than perfectly calibrated long-term uncertainty bounds.
