# Supplementary material for: Forecasting continued increase in primary and secondary syphilis in the United States, 2024–2033: a state-level multi-model ensemble study
Source: Front Public Health. 2026 Jul 24;14:1888674. doi: 10.3389/fpubh.2026.1888674 (PMC13447349; doi:10.3389/fpubh.2026.1888674)
Supplement: Supplementary file 3 [file Data_Sheet_3.DOCX]

**Supplementary Text S3. Predictor Timing, Information Boundaries, and Leakage Audit**

To make the temporal logic of the forecasting pipeline auditable, we summarized each predictor’s source, lag structure, timing of availability, and observed-versus-extrapolated status (Table 1- Table 3), and we separately summarized the information boundary used at each forecasting stage (see Table 3). The audit shows an important distinction between the outcome-derived syphilis history features and the exogenous covariate or population inputs.

For the outcome-derived syphilis history variables, the pipeline is strictly backward-looking. Specifically, lag1_log_rate is defined from year t-1, rolling3_log_rate from years t-1 to t-3, and lag1_delta_log_rate from the difference between years t-1 and t-2. The contemporaneous year-t outcome is therefore not used to predict year t. During recursive forecasting, once a prediction for year t is produced, that prediction is written back into the syphilis history and becomes available for constructing the lagged syphilis features used at year t+1. This logic is used in the external holdout evaluation, future forecasting, and rolling-origin backtesting.

The exogenous covariates follow a different pattern. Gonorrhea, chlamydia, HIV, poverty, unemployment, and income predictors enter the models only through lagged values or lagged annual changes, so their formulas are also backward-looking. However, in the current implementation the retrospective holdout and rolling-origin evaluations do not impose an origin-specific truncation on the exogenous covariate panel or on the population panel. As a result, when the pipeline retrospectively forecasts years up to 2023, it still accesses realized post-origin covariate and population values for those target years. By contrast, the 2024-2033 future forecasts use extrapolated covariate trajectories and extrapolated population values rather than future observed values.

Table 1 Predictor timing and leakage audit for core, population, and syphilis-history predictors

| **Feature group** | **Feature** | **Used in** | **Definition for prediction year *t*** | **Availability timing** | **Historical rows 2003–2023** | **Holdout / rolling-origin evaluation** | **Future 2024–2033 forecasts** | **Leakage audit note** | **Primary verification files** |
| --- | --- | --- | --- | --- | --- | --- | --- | --- | --- |
| Core time index | year_idx | minimal + enhanced + Bayesian | year_idx = t − 2000 | Known at year *t* | Observed metadata only | Observed metadata only | Observed metadata only | No dependence on outcomes or future covariates | forecast_pipeline.py; processed_panel.csv; run_config.json |
| Geographic identifier | area_id | XGBoost + NN categorical input | Static jurisdiction identifier | Fixed before prediction | Observed metadata only | Observed metadata only | Observed metadata only | Static identifier only | forecast_pipeline.py; processed_panel.csv; observed_panel_with_national.csv |
| National-reference flag | is_national | minimal + enhanced fixed effects and diagnostics | 1 if area_id = 00, else 0 | Fixed before prediction | Observed metadata only | Observed metadata only | Observed metadata only | Code-generated structural flag | forecast_pipeline.py; processed_panel_with_national_reference.csv; observed_panel_with_national.csv |
| Population level | population | minimal + enhanced + Bayesian offset/input | Population for year *t* | Must be available at year *t* | Observed population for year *t* | Uses realized population for post-origin years ≤ 2023 | Uses extrapolated population for 2024–2033 | No target-outcome leakage, but strict real-time availability after origin is not enforced in retrospective evaluation | forecast_pipeline.py; population_observed_and_extrapolated.csv; raw population input |
| Population transform | log_population | minimal + enhanced non-Bayesian fixed effect only | log(population_t) | Must be available at year *t* | Observed population transformed on the same row | Uses realized population for post-origin years ≤ 2023 | Uses extrapolated population for 2024–2033 | Removed from Bayesian fixed effects because population enters through the offset | forecast_pipeline.py; population_observed_and_extrapolated.csv; processed_panel.csv |
| Syphilis history | lag1_log_rate | minimal + enhanced + Bayesian | log(1 + rate_{t−1}) | Available only after year *t−1* closes | Observed lag from *t−1* only | Starts from observed history through the origin, then uses prior predictions as forecasting moves forward | 2024 uses observed 2023; later years use previously predicted syphilis history as needed | Strictly uses *t−1* only; year *t* outcome is never used as a predictor | forecast_pipeline.py; observed_panel_with_national.csv; processed_panel.csv; holdout_predictions.csv; future_forecasts_2024_2033.csv |
| Syphilis history | rolling3_log_rate | minimal + enhanced + Bayesian | Mean of log(1 + rate) at *t−1* to *t−3* | Available only after years *t−1* to *t−3* close | Observed lag window only | Starts from observed history through the origin, then uses prior predictions as forecasting moves forward | Recursive from prior observed or predicted syphilis history only | Strictly backward-looking 3-year rolling mean; year *t* is never included | forecast_pipeline.py; observed_panel_with_national.csv; processed_panel.csv |
| Syphilis history | lag1_delta_log_rate | minimal + enhanced + Bayesian | log(1 + rate_{t−1}) − log(1 + rate_{t−2}) | Available only after years *t−1* and *t−2* close | Observed lagged change only | Starts from observed history through the origin, then uses prior predictions as forecasting moves forward | Recursive from prior observed or predicted syphilis history only | Lagged annual change only; year *t* outcome is not used | forecast_pipeline.py; observed_panel_with_national.csv; processed_panel.csv |

Table 2 Predictor Timing, Availability, and Leakage Audit for Exogenous Covariates

| **Feature group** | **Feature** | **Used in** | **Definition for prediction year *t*** | **Availability timing** | **Historical rows 2003–2023** | **Holdout / rolling-origin evaluation** | **Future 2024–2033 forecasts** | **Leakage audit note** | **Primary verification files** |
| --- | --- | --- | --- | --- | --- | --- | --- | --- | --- |
| Related STI covariate | lag1_gonorrhea_log_rate | enhanced + Bayesian enhanced | log(1 + gonorrhea rate_{t−1}) | Available after year *t−1* closes | Observed lag from historical covariate panel | Uses realized post-origin covariate values for years ≤ 2023 | Uses extrapolated gonorrhea series for 2024–2033 | Formula is strictly lagged, but origin-specific truncation is not enforced retrospectively | forecast_pipeline.py; covariate_panel_observed_and_extrapolated.csv; raw gonorrhea file |
| Related STI covariate | rolling3_gonorrhea_log_rate | enhanced + Bayesian enhanced | Mean of gonorrhea log-rates at *t−1* to *t−3* | Available after years *t−1* to *t−3* close | Observed lag window | Uses realized post-origin covariate values for years ≤ 2023 | Uses extrapolated gonorrhea series for 2024–2033 | Backward-looking in formula, but retrospective evaluation still accesses realized post-origin rows | Same as above |
| Related STI covariate | lag1_chlamydia_log_rate | enhanced + Bayesian enhanced | log(1 + chlamydia rate_{t−1}) | Available after year *t−1* closes | Observed lag | Uses realized post-origin covariate values for years ≤ 2023 | Uses extrapolated chlamydia series for 2024–2033 | Same issue as above | forecast_pipeline.py; covariate_panel_observed_and_extrapolated.csv; raw chlamydia file |
| Related STI covariate | rolling3_chlamydia_log_rate | enhanced + Bayesian enhanced | Mean of chlamydia log-rates at *t−1* to *t−3* | Available after years *t−1* to *t−3* close | Observed lag window | Uses realized post-origin covariate values for years ≤ 2023 | Uses extrapolated chlamydia series for 2024–2033 | Same issue as above | Same as above |
| Socioeconomic covariate | lag1_poverty_rate | enhanced + Bayesian enhanced | poverty rate_{t−1} | Available after year *t−1* closes | Observed lag | Uses realized post-origin covariate values for years ≤ 2023 | Uses extrapolated poverty series for 2024–2033 | Strictly lagged in formula, but not origin-truncated retrospectively | forecast_pipeline.py; covariate_panel_observed_and_extrapolated.csv; raw SAIPE file |
| Socioeconomic covariate | lag1_unemployment_rate | enhanced + Bayesian enhanced | unemployment rate_{t−1} | Available after year *t−1* closes | Observed lag | Uses realized post-origin covariate values for years ≤ 2023 | Uses extrapolated unemployment series for 2024–2033 | Same issue as above | forecast_pipeline.py; covariate_panel_observed_and_extrapolated.csv; raw BLS file |
| Socioeconomic covariate | lag1_log_median_income | enhanced + Bayesian enhanced | log(median household income_{t−1}) | Available after year *t−1* closes | Observed lag | Uses realized post-origin covariate values for years ≤ 2023 | Uses extrapolated income series for 2024–2033 | Same issue as above | forecast_pipeline.py; covariate_panel_observed_and_extrapolated.csv; raw SAIPE file |
| Socioeconomic change | lag1_poverty_delta | enhanced + Bayesian enhanced | poverty rate_{t−1} − poverty rate_{t−2} | Available after years *t−1* and *t−2* close | Observed lagged change | Uses realized post-origin covariate values for years ≤ 2023 | Uses extrapolated poverty series for 2024–2033 | Lagged in formula, but retrospective evaluation still accesses realized post-origin rows | Same as above |
| Socioeconomic change | lag1_unemployment_delta | enhanced + Bayesian enhanced | unemployment rate_{t−1} − unemployment rate_{t−2} | Available after years *t−1* and *t−2* close | Observed lagged change | Uses realized post-origin covariate values for years ≤ 2023 | Uses extrapolated unemployment series for 2024–2033 | Same issue as above | Same as above |
| Socioeconomic change | lag1_income_log_delta | enhanced + Bayesian enhanced | log(income_{t−1}) − log(income_{t−2}) | Available after years *t−1* and *t−2* close | Observed lagged change | Uses realized post-origin covariate values for years ≤ 2023 | Uses extrapolated income series for 2024–2033 | Same issue as above | Same as above |
| HIV covariate | lag1_hiv_log_rate | enhanced + Bayesian enhanced | log(1 + HIV rate_{t−1}) if observed | Available after year *t−1* closes | Observed lag where available | Uses realized post-origin covariate values for years ≤ 2023 | Uses extrapolated HIV series for 2024–2033 where available | Strictly lagged in formula, but retrospective evaluation still accesses realized post-origin rows | forecast_pipeline.py; covariate_panel_observed_and_extrapolated.csv; raw HIV file |
| HIV covariate | rolling3_hiv_log_rate | enhanced + Bayesian enhanced | Mean of available HIV log-rates at *t−1* to *t−3* | Available after years *t−1* to *t−3* close | Observed lag window where available | Uses realized post-origin covariate values for years ≤ 2023 | Uses extrapolated HIV series for 2024–2033 where available | Same issue as above | Same as above |
| HIV missingness flag | lag1_hiv_missing | enhanced + Bayesian enhanced | 1 if HIV log-rate at *t−1* is missing | Available once the *t−1* field is observed or known missing | Observed from historical missingness pattern | Uses realized post-origin missingness pattern for years ≤ 2023 | Uses extrapolated-panel missingness pattern for 2024–2033 | Represents lagged missingness status, but still references realized post-origin rows retrospectively | Same as above |
| HIV missingness flag | rolling3_hiv_missing_share | enhanced + Bayesian enhanced | Share of missing HIV values across *t−1* to *t−3* | Available once the *t−1* to *t−3* fields are observed or known missing | Observed from historical missingness pattern | Uses realized post-origin missingness pattern for years ≤ 2023 | Uses extrapolated-panel missingness pattern for 2024–2033 | Same issue as above | Same as above |

Table 3 Information-Boundary Audit by Forecasting Stage

| **Pipeline stage** | **Target years** | **Model fit data available at stage** | **Syphilis history used to build features** | **Covariates used to build features** | **Population used to build features** | **Uses actual target-year outcome as predictor** | **Observed or forecasted inputs** | **Interpretation under strict no-leakage audit** | **Primary verification files** |
| --- | --- | --- | --- | --- | --- | --- | --- | --- | --- |
| Historical feature construction for model frame | 2003–2023 | Observed years only up to each row’s year *t* | Observed syphilis rates from *t−1*, *t−2*, *t−3* only | Observed covariates from *t−1* or *t−1:t−3* only | Observed population for year *t* | No | Observed historical inputs only | Feature construction within the historical panel is time-causal row by row | forecast_pipeline.py; processed_panel.csv; processed_panel_with_national_reference.csv; observed_panel_with_national.csv; covariate_panel_observed_and_extrapolated.csv; population_observed_and_extrapolated.csv |
| Internal conformal calibration run | 2017–2018 | Stage model fit on 2003–2016 only | Recursive forecasts start from observed history through 2016, then update history with predicted 2017 before forecasting 2018 | Uses realized covariate values for 2016–2017 lags because the covariate panel is not truncated at the 2016 origin | Uses realized population for 2017–2018 | No | Predicted syphilis path plus realized post-origin exogenous inputs | No leakage of target outcome itself, but strict origin-specific availability is not enforced for exogenous covariates or population | forecast_pipeline.py; run_config.json; holdout_predictions.csv; covariate_panel_observed_and_extrapolated.csv; population_observed_and_extrapolated.csv |
| External holdout recursive forecast | 2019–2023 | Final model fit on 2003–2018 only | Recursive forecasts start from observed history through 2018; predicted 2019 is then fed forward to generate 2020, and so on | Uses realized covariate values for years ≤ 2023, including post-origin lagged covariates during retrospective holdout evaluation | Uses realized population for 2019–2023 | No | Predicted syphilis path plus realized post-origin exogenous inputs | Outcome-history leakage is avoided, but strict real-time no-leakage is not satisfied for exogenous covariates or population in retrospective holdout evaluation | forecast_pipeline.py; holdout_predictions.csv; observed_panel_with_national.csv; covariate_panel_observed_and_extrapolated.csv; population_observed_and_extrapolated.csv |
| Future recursive forecast | 2024–2033 | Same final model fit on 2003–2018 | Recursive forecasts continue the predicted syphilis path forward; future lagged syphilis features are based on prior predictions once observed history is exhausted | Uses extrapolated covariate values from the future covariate panel | Uses extrapolated population for 2024–2033 | No | Predicted syphilis path plus extrapolated exogenous inputs | No future observed outcome or future observed covariate is introduced in the 2024–2033 stage | forecast_pipeline.py; future_forecasts_2024_2033.csv; covariate_panel_observed_and_extrapolated.csv; population_observed_and_extrapolated.csv |
| Rolling-origin backtesting | Origin+1 to min(origin+3, 2023) for origins 2012, 2014, 2016, 2018 | Each model is re-fit using only years 2003–origin | Recursive forecasts start from observed history through the origin year and then feed predictions forward within the 1–3 year horizon | Uses realized covariate values for post-origin years ≤ 2023 because the covariate panel is not truncated at each origin | Uses realized population for backtest target years | No | Predicted syphilis path plus realized post-origin exogenous inputs | Outcome-history leakage is avoided, but strict origin-specific no-leakage is not satisfied for exogenous covariates or population in retrospective backtesting | forecast_pipeline.py; rolling_origin_predictions.csv; rolling_origin_metrics.csv; covariate_panel_observed_and_extrapolated.csv; population_observed_and_extrapolated.csv |
| National aggregate output | 2019–2033 and rolling-origin national summaries | No separate national model is fit | State-level predictions are generated first, then summed to create national forecasts | State-level covariates follow the same realized or extrapolated rules as above | National population is the sum of state populations for each year | No | Derived from state-level predictions only | National outputs do not create an additional leakage path; they inherit the same information boundary as the state-level stage from which they are aggregated | Not specified |
