# Supplementary material for: Forecasting continued increase in primary and secondary syphilis in the United States, 2024–2033: a state-level multi-model ensemble study
Source: Front Public Health. 2026 Jul 24;14:1888674. doi: 10.3389/fpubh.2026.1888674 (PMC13447349; doi:10.3389/fpubh.2026.1888674)
Supplement: Supplementary file 4 [file Data_Sheet_4.DOCX]

**Supplementary Text S4. Evidence-Based Exploration of the Poor Performance of the Bayesian Negative Binomial Model**

*This text summarizes direct evidence from the latest code and output files and then provides clearly labeled evidence-based interpretations.*

# 1. Scope and Overall Conclusion

This supplementary text examines why the Bayesian negative binomial model performed substantially worse than the other candidate models in the present state-level syphilis forecasting study. The goal is not to make a general claim about Bayesian forecasting methods as a whole, but rather to evaluate the behavior of the specific Bayesian negative binomial BYM2/CAR implementation used here under a long-horizon recursive forecasting design.

Directly from the official external-holdout scoring file, the Bayesian model was the worst candidate model in both feature sets. Its holdout WAPE was 65.60% in the enhanced specification and 74.31% in the minimal specification, compared with 19.98% for enhanced XGBoost, 24.63% for enhanced neural network, 22.70% for minimal XGBoost, and 26.42% for minimal neural network. Its mean bias was also strongly positive (+501.92 cases for enhanced and +610.37 for minimal), indicating systematic upward error rather than balanced noise.

The overall conclusion is therefore not simply that one prior was mis-specified. Instead, the evidence is more consistent with a structural mismatch between the current Bayesian implementation and the present forecasting setting, which combines heterogeneous state-level annual panel data with long-horizon recursive extrapolation. The minimal Bayesian specification also showed additional sampling instability: it had 3 divergences, 45 of 61 parameters with R-hat > 1.01, and 36 of 61 parameters with bulk ESS < 100, whereas the enhanced specification had 0 divergences and only 3 of 77 parameters with bulk ESS below 400.

# 2. Direct Evidence That the Problem Was Not Resolved by Prior Tuning

Prior-sensitivity analyses showed that the Bayesian model remained poor under all tested prior alternatives. In the enhanced specification, the main-prior holdout WAPE was 65.60%. Under the lag-1 alternatives it became 66.76% and 67.12%; under the rolling-3 alternatives it became 66.67% and 67.20%; under the rho alternatives it became 66.83% and 67.27%; and under the alternative spatial_alpha prior it became 66.68%. Thus, every tested enhanced sensitivity run remained more than 46 percentage points worse than enhanced XGBoost (19.98%) and more than 40 percentage points worse than enhanced neural network (24.63%).

In the minimal specification, the main-prior holdout WAPE was 74.31%. Under the lag-1 alternatives it became 72.38% and 72.75%; under the rolling-3 alternatives it became 71.69% and 73.85%; under the rho alternatives it became 73.45% and 73.58%; and under the alternative spatial_alpha prior it became 74.48%. These values show that plausible prior changes could move performance modestly, but even the best minimal sensitivity result (71.69%) still remained dramatically worse than the stronger non-Bayesian models.

Taken together, these results are direct evidence that the poor performance was not rescued by plausible changes to the main informative or regularizing priors. This argues against the explanation that one isolated prior setting was solely responsible for the model failure.

# 3. Direct Evidence From Posterior Sampling Diagnostics

The minimal Bayesian specification showed clear sampling problems. The diagnostic summary reported 3 divergences, a maximum R-hat of 1.09, and minimum bulk and tail ESS values of 37 and 37. Several key parameters had weak mixing, including spatial_alpha (mean 0.807, 95% HDI 0.326 to 1.000, R-hat = 1.09, ESS bulk = 37), beta:year_idx (mean 0.173, 95% HDI 0.079 to 0.240, R-hat = 1.08, ESS bulk = 44), sigma_u (mean 0.321, 95% HDI 0.027 to 0.525, R-hat = 1.07, ESS bulk = 49), and beta:rolling3_log_rate (R-hat = 1.07, ESS bulk = 50).

By contrast, the enhanced Bayesian specification was much cleaner from a sampling perspective. It showed 0 divergences, a maximum R-hat of 1.05, and minimum bulk and tail ESS values of 144 and 141. Its main lag coefficient beta:lag1_log_rate was well mixed (posterior mean 0.619, 95% HDI 0.485 to 0.758, ESS bulk = 1851, R-hat = 1.00). Enhanced rho and spatial_alpha were not perfectly stable, but were still much better than in the minimal model: rho = 0.533 (95% HDI 0.000 to 0.933, R-hat = 1.05, ESS bulk = 144) and spatial_alpha = 0.662 (95% HDI 0.114 to 1.000, R-hat = 1.02, ESS bulk = 260).

These diagnostic contrasts matter because the enhanced Bayesian model still had very poor holdout performance (WAPE = 65.60%) despite its much cleaner sampling behavior. This shows that weak predictive performance cannot be explained by convergence problems alone. Instead, the minimal model appears to combine the same forecasting failure with additional posterior sampling instability.

# 4. Direct Evidence From Holdout Prediction Behavior

The official holdout scoring was based on 255 state/DC rows from 2019 to 2023, excluding the separate national_state_sum rows. On these 255 scored observations, Bayesian errors were not merely noisy or randomly distributed. Instead, they became progressively worse over time and were predominantly upward. At the state level, annual WAPE for the enhanced Bayesian model increased from 0.161 in 2019 to 0.328 in 2020, 0.431 in 2021, 0.717 in 2022, and 1.439 in 2023. The minimal Bayesian model showed the same pattern, increasing from 0.189 in 2019 to 0.427 in 2020, 0.525 in 2021, 0.804 in 2022, and 1.554 in 2023.

Across all 255 state/DC holdout rows, the enhanced Bayesian model overpredicted in 57.65% of observations and had a median signed error of +16.0 cases, a mean bias of +501.92 cases, and a 95th-percentile signed error of +2374.6 cases. The minimal Bayesian model overpredicted in 60.00% of observations and had a median signed error of +21.0 cases, a mean bias of +610.37 cases, and a 95th-percentile signed error of +3096.35 cases. These values show a strongly right-skewed upward error pattern rather than symmetric noise around zero.

By 2023, the state-level total predicted cases were already far above the observed totals. For the enhanced Bayesian model, the summed state prediction reached 125,216 compared with 53,007 observed cases, or about 2.36 times the observed total. For the minimal Bayesian model, the summed state prediction reached 130,992 compared with 53,007 observed cases, or about 2.47 times the observed total. The upward drift also became more widespread over time: in 2023, 70.59% of enhanced state predictions and 72.55% of minimal state predictions exceeded the observed state counts.

The largest state-level misses were strongly upward rather than balanced around zero. For example, in 2023 the enhanced Bayesian model predicted 34,973 cases in California versus 6,348 observed, and 13,676 in New York versus 2,887 observed. The minimal Bayesian model predicted 13,007 cases in Florida versus 4,366 observed. These patterns indicate systematic upward drift rather than isolated local mistakes.

# 5. Direct Evidence From Future Forecast Trajectories

The future forecasts suggest that the upward problem was already established at the moment the model entered the true future period. The observed national total in 2023 was 53,007 cases. Yet the Bayesian national_state_sum forecast for 2024 was already 181,346.0 in the enhanced specification and 183,398.5 in the minimal specification, corresponding to about 3.42 times and 3.46 times the 2023 observed total, respectively.

By 2033, the national_state_sum Bayesian forecasts had become extremely large: 2,891,359.5 in the enhanced specification and 4,594,987.5 in the minimal specification. Relative to the 2023 observed national total, these correspond to approximately 54.55 times and 86.69 times the observed level. Relative to their own 2024 Bayesian starting points, they imply 15.94-fold and 25.05-fold growth from 2024 to 2033.

These trajectories were far above the corresponding non-Bayesian national forecasts. For example, enhanced XGBoost predicted 52,700.7 cases in 2024 and 99,105.0 in 2033, while enhanced neural network predicted 66,485.9 in 2024 and 144,377.5 in 2033. This contrast supports the interpretation that the Bayesian trajectory was not simply reflecting the overall difficulty of the task, but was following a qualitatively different and much more explosive path.

This pattern is important because it suggests that the main failure mechanism had already emerged before remote future covariate extrapolation could dominate the trajectory. In other words, the model was already on an unrealistically elevated path at the very start of the future forecasting period.

# 6. Direct Evidence From Probabilistic Performance

The Bayesian model also performed poorly as a probabilistic forecaster. In the enhanced specification, mean CRPS was 486.95, PIT mean was 0.4399, PIT SD was 0.3528, and the PIT Kolmogorov-Smirnov p-value was 5.48e-07. In the minimal specification, mean CRPS was 535.65, PIT mean was 0.4451, PIT SD was 0.3551, and the PIT Kolmogorov-Smirnov p-value was 9.08e-06.

Empirical interval coverage was also poor. In the enhanced Bayesian model, nominal 80% intervals covered only 150 of 255 state/DC holdout observations (58.82%), and nominal 95% intervals covered only 191 of 255 observations (74.90%). In the minimal Bayesian model, nominal 80% intervals covered 148 of 255 observations (58.04%), and nominal 95% intervals covered 186 of 255 observations (72.94%). These values are far below nominal levels.

The Bayesian probabilistic results were also much worse than those of the stronger competing models. For example, enhanced XGBoost had mean CRPS 137.45, and the final top-3 ensemble had mean CRPS 149.03. These contrasts show that the problem was not limited to point forecasts. The posterior predictive distributions were also poorly calibrated, indicating that the model failed both in central tendency and in uncertainty representation.

# 7. Evidence-Based Interpretation of the Most Likely Failure Mechanism

Inspection of the current implementation provides direct structural context for these empirical patterns. The Bayesian model was fit once on the 2003-2018 fitting data and was then used to generate a fully recursive forecast path from 2019 onward. After each year was predicted, the predicted value was written back into the syphilis history and used to construct the lagged syphilis predictors for the next year. Thus, by 2033 the model was relying on a long chain of model-generated rather than observed syphilis history.

The posterior summaries show that the lag-1 syphilis term remained strongly positive in both Bayesian specifications. The posterior mean of beta:lag1_log_rate was 0.619 in the enhanced model and 0.634 in the minimal model, with 95% HDIs of 0.485 to 0.758 and 0.496 to 0.766, respectively. In the enhanced model, additional lagged covariate effects were also clearly positive for some terms, including beta:lag1_log_median_income = 0.300 (95% HDI 0.203 to 0.410) and beta:lag1_poverty_rate = 0.292 (95% HDI 0.191 to 0.383). The minimal model also showed a clearly positive year trend, with beta:year_idx = 0.173 (95% HDI 0.079 to 0.240), whereas the enhanced year trend was smaller and crossed zero (0.085, 95% HDI -0.020 to 0.207).

The spatial hyperparameters also suggest that the model was not operating in a weakly structured regime. In the enhanced model, rho was 0.533 and spatial_alpha was 0.662; in the minimal model, rho was 0.856 and spatial_alpha was 0.807. The code-generated posterior-risk files also flagged the main lag term as a recursive amplification risk, with explosion_risk_score = 1.238 in the enhanced model and 1.268 in the minimal model.

These are direct empirical facts. Based on them, the most plausible inference is that the recursive forecasting structure allowed positive lag memory, positive trend pressure, and some positive lagged covariate effects to reinforce one another over time. Because the model was fit on the log scale for the negative binomial mean and also included a population offset and time-invariant BYM2/CAR area effects, even moderate upward bias in the latent linear predictor could be repeatedly carried forward and translated into rapidly growing counts. This interpretation is further supported by the pipeline's own posterior-risk annotations, which explicitly flag positive year effects, strong lag persistence, positive lagged covariate effects, and large area effects as features that can amplify recursive forecasts.

A further evidence-based interpretation concerns the distinction between the two Bayesian specifications. The enhanced model appears to represent a primarily structural failure: it sampled relatively cleanly, yet still produced strong upward drift and poor calibration. The minimal model appears to combine the same structural problem with additional posterior sampling instability, which likely made its forecasts even less reliable.

# 8. Boundary of Interpretation

These results should not be interpreted as evidence that Bayesian forecasting is inherently unsuitable for infectious-disease prediction. Rather, they indicate that the specific Bayesian negative binomial BYM2/CAR implementation evaluated here did not match the present forecasting problem well. The current setting involved annual state-level panel data, substantial cross-state heterogeneity, strong recent upward trends, and a long recursive extrapolation path extending from a 2003-2018 fit through 2019-2033 forecasting.

Accordingly, the Bayesian results in this study are best interpreted as an empirical caution about this particular model-task combination, rather than as a general rejection of Bayesian approaches. The key empirical point is that this implementation remained poor under all tested prior alternatives, produced WAPE values of 65.60% and 74.31%, and showed severe upward drift even before the distant future horizons were reached.
